# Natural genetic variation as a tool for discovery in *Caenorhabditis* nematodes

**DOI:** 10.1093/genetics/iyab156

**Published:** 2022-01-04

**Authors:** Erik C Andersen, Matthew V Rockman

**Affiliations:** 1 Department of Molecular Biosciences, Northwestern University, Evanston, IL 60201, USA; 2 Department of Biology and Center for Genomics & Systems Biology, New York University, New York, NY 10003, USA

**Keywords:** *Caenorhabditis*, quantitative genetics, QTL mapping, recombinant inbred lines, genetic variation, WormBook

## Abstract

Over the last 20 years, studies of *Caenorhabditis elegans* natural diversity have demonstrated the power of quantitative genetic approaches to reveal the evolutionary, ecological, and genetic factors that shape traits. These studies complement the use of the laboratory-adapted strain N2 and enable additional discoveries not possible using only one genetic background. In this chapter, we describe how to perform quantitative genetic studies in *Caenorhabditis*, with an emphasis on *C. elegans*. These approaches use correlations between genotype and phenotype across populations of genetically diverse individuals to discover the genetic causes of phenotypic variation. We present methods that use linkage, near-isogenic lines, association, and bulk-segregant mapping, and we describe the advantages and disadvantages of each approach. The power of *C. elegans* quantitative genetic mapping is best shown in the ability to connect phenotypic differences to specific genes and variants. We will present methods to narrow genomic regions to candidate genes and then tests to identify the gene or variant involved in a quantitative trait. The same features that make *C. elegans* a preeminent experimental model animal contribute to its exceptional value as a tool to understand natural phenotypic variation.

## Introduction 

Natural genetic variation is evolution’s raw material and provides a window into the processes that shape the living world. It is also a big collection of mutant alleles. Among the hundreds of thousands of sites that differ between two wild isolates of *Caenorhabditis* *elegans* are alleles that perturb your favorite molecular pathway, pushing a biochemical equilibrium slightly beyond where it sits in the N2 strain, generating worms that are measurably different in phenotype. These natural mutations stand ready to lead us from phenotype to molecular gene. A simple method, quantitative genetic mapping, provides a way to use natural genetic variation to discover molecular functions and shed light on evolutionary patterns and processes.

The field of quantitative genetics has its roots in the early days of human biometrics and agricultural genetics, where it focused on variation in quantitative traits, which are measured on a continuous scale. Over time, as geneticists discovered that quantitative variation is just another flavor of Mendelian genetics (Fisher 1918), and that even discrete traits, like disease status, result from genetic risk factors that themselves vary quantitatively, the name quantitative genetics became attached to the broader field of natural genetic variation. We use the name in this modern sense, to refer to studies that investigate the effects of natural genetic variation, as opposed to studies that use laboratory-generated alleles or perturbations. For many molecular biologists, quantitative genetics is an opaque and esoteric field, where a priesthood of statisticians offers auguries from behind a veil of LOD scores, LD statistics, negative-log-10-*P*-values, and genomic relationship matrices. Our goal here is to demystify quantitative genetics and illustrate its enormous utility for research in *Caenorhabditis* nematodes, with a focus on *C. elegans* and its selfing relatives, *Caenorhabditis briggsae* and *Caenorhabditis* *tropicalis*.

To start, we will set aside the statistical complexities and focus on the core elements of *C. elegans* quantitative trait locus (QTL) mapping, the quantitative genetics version of forward genetics, and the method most useful to researchers interested in using natural variation to discover new molecular players in their corners of worm biology. The goal of QTL mapping is to identify a region of the genome that contains a locus (a QTL) whose alleles affect a quantitative measurement of a phenotype. For our purposes, QTL mapping includes a range of techniques, including both linkage and genome-wide association (GWA) mapping studies, and they all share the following framework in three simple steps. First, we require a collection of genetically distinct worm strains that differ from one another phenotypically because of the unique combinations of variants each strain carries. Such variation is almost always present among wild worm strains or among recombinant strains derived from them, for literally any trait. Pumping rate? Yes. Penetrance of some phenotype after a perturbation? Yes. Probability that a particular serine is phosphorylated in a protein expressed in the ASEL neuron during the early L4 stage? The answer will be yes, and the challenge is simply to measure it. Second, we require genotype data for the worm strains. The number of positions whose genotypes we require varies by mapping technique, with some methods needing only a handful of positions per chromosome and others needing dense genome-wide data, but in *C. elegans* and its relatives, most of the genotyping work has already been done, and one need only order the strains. Of course, one of the great virtues of *C. elegans* for quantitative genetics is that we start with inbred lines, which can be genotyped once and phenotyped forever. Third, we perform a statistical test, where the priesthood historically steps in and asks the molecular biologists to shield their eyes. But the man behind the curtain is just a test for a difference between two means, the kind of test that molecular geneticists perform in every paper. We simply ask, does the average phenotype differ between those strains that are homozygous for allele 1 at this position and those strains that are homozygous for allele 2? For a continuous trait, we can use a *t*-test. For a binary trait, we can use a chi-square test for independence. For a trait with a weird distribution, we can use a nonparametric test, which simply converts the phenotype values to ranks and then asks whether the ranks differ between the two genotypic classes. Statisticians will have opinions about which tests are best in which settings, and often an optimal test exists from the perspective of statistical power. In the sections below about specific experimental designs, we provide some recommendations. But conceptually—and practically, in almost all cases—the choice of test is inconsequential.

A good way to understand QTL mapping is to think about it as an example of a broader class of experimental design, a *randomized multifactorial perturbation*. When we sample wild isolates of *C. elegans*, or recombinant lines derived from them, they differ at a large number of genetic positions. Each of these genetic differences represents a perturbation, a mutation that may (or may not) alter the animal’s biology. In conventional forward genetics, we aim to study strains that carry one perturbation at a time. By comparing mutant to wild-type, we can test for an effect of that single perturbation. If we detect a difference, we can attribute it to the single mutation that differs between strains (notwithstanding background mutations—always outcross your mutants!). QTL mapping departs from that approach by including huge numbers of perturbations all at once—hence, multifactorial perturbation. Now, when two strains differ in phenotype, we are unable to point to any single mutation as the causal variant. The key to discovering the causal variants is to examine each perturbation while shuffling all of the other perturbations—randomizing them. If the randomization is effective, all the other perturbations just add a bit of random variation, and all of the systematic variation will be caused by the focal perturbation. Consider a collection of inbred *C. elegans* strains in which every variant is randomized with respect to every other variant (equivalently, they are uncorrelated, so knowing the genotype at one position provides no information about the genotype at another position). After measuring the phenotypes of these inbred lines, we can take each variant, one at a time, and test whether the two classes of homozygotes have different average phenotypes. We can then march through the genome, testing every position—idealized QTL mapping.

**Figure 1:**
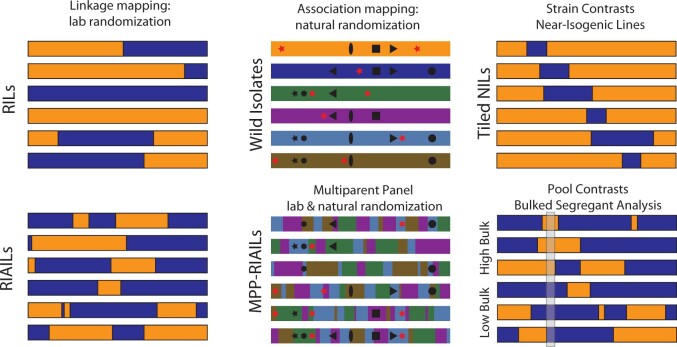
QTL-mapping panels. Different experimental designs make different compromises along many axes, including detection power, mapping resolution, and genetic diversity. Laboratory crosses of a pair of strains provide a straightforward route to mapping by linkage in RILs, represented here as a single chromosome from each of six RILs from a cross of two strains, one with an orange genome and the other blue. Because of the low recombination rate in *C. elegans*, RIL chromosomes have an average of one crossover each. By adding generations of intercrossing prior to inbreeding, RIAILs increase the number of breakpoints, increasing mapping resolution. Association mapping uses historical recombination to randomize alleles. Wild isolates carry a mixture of common alleles that arose in ancient ancestors (black symbols) and more recent alleles that are unique to each strain (red stars). The pattern of association among shared variants (LD) is governed by the population history of recombination, and some variants may be perfectly correlated (*e.g*., black star and black hexagon). Multiparent panels use laboratory crosses to shuffle wild isolate genomes even more, reducing LD and increasing the frequency of rare alleles; now even singletons (red stars) are visible to QTL mapping. QTL can also be discovered by comparing strains that differ only in a small interval—near-isogenic lines. Finally, bulk segregant and related evolve-and-resequence methods do away with the construction of inbred lines. They detect QTL as differences in allele frequencies between pools of individuals selected to differ in phenotype. In the figure, the allele frequencies differ between the high-phenotype pool and the low-phenotype pool in the highlighted interval.

In practice, the major challenge of QTL mapping as a randomized multifactorial perturbation is the randomization step: how can we get all of the perturbations to be independent of one another? Population geneticists refer to correlations among loci as linkage disequilibrium (LD), and much of the experimental and statistical work of QTL mapping centers on reducing or controlling LD to achieve effective randomization. In this paper, we focus on a handful of experimental designs for mapping panels, collections of strains whose patterns of variation and LD make them useful for QTL mapping, represented schematically in Figure 1.

## Screens leave genes on the table

Genetic screens are one of our most powerful methods for biological discovery. Quantitative genetics, like a mutagenesis screen, can point us to genes with dramatic effects on phenotypes and processes of interest. In addition, quantitative genetics can reveal a broader spectrum of alleles and can point to genes that other methods would struggle to find or would miss altogether. We can point to at least four classes of discovery where quantitative genetic methods excel.

First, quantitative genetics can reveal mutations whose effects are modest in size or whose phenotypes have low penetrance. In a classical mutagenesis screen, the number of homozygous mutations scored for each mutant phenotype is typically low, limited by time and resources required to examine each animal, and the scoring is typically qualitative. If the effect of a mutation is small or manifests only a fraction of the time, a screen might not identify it. Consider in contrast a quantitative genetic experiment that scores 100 strains, each carrying a unique mosaic of alleles sampled from two inbred wild strains (as in Figure 1, RILs and RIAILs). Now, for the cost of phenotyping 100 strains, *every* mutation will be assayed on average 50 times (each parent allele is homozygous in approximately half of the 100 offspring). Randomized multifactorial perturbation provides high levels of replication at the level of mutation without requiring high levels of replication at the level of strain. This means that quantitative genetics can find genes of small effect or incomplete penetrance, but equivalently it means that the method allows for studies of noisier phenotypes, or for lower-resolution higher-throughput phenotyping at the screening stage of analysis. In *C. elegans*, the built-in replication of randomized multifactorial perturbation has proven itself in studies of traits that are laborious, slow, and expensive to score (*e.g*., Ghosh *et al.* 2012a, 2012b; Farhadifar *et al.* 2020); traits that are low penetrance (*e.g*., Noble *et al.* 2015); traits that are noisy and sensitive to random environmental variation (Bendesky *et al.* 2011, 2012); and traits that can be screened using high-throughput assays (Andersen *et al.* 2015; Zdraljevic *et al.* 2017; Evans *et al.* 2020).

**Figure 2:**
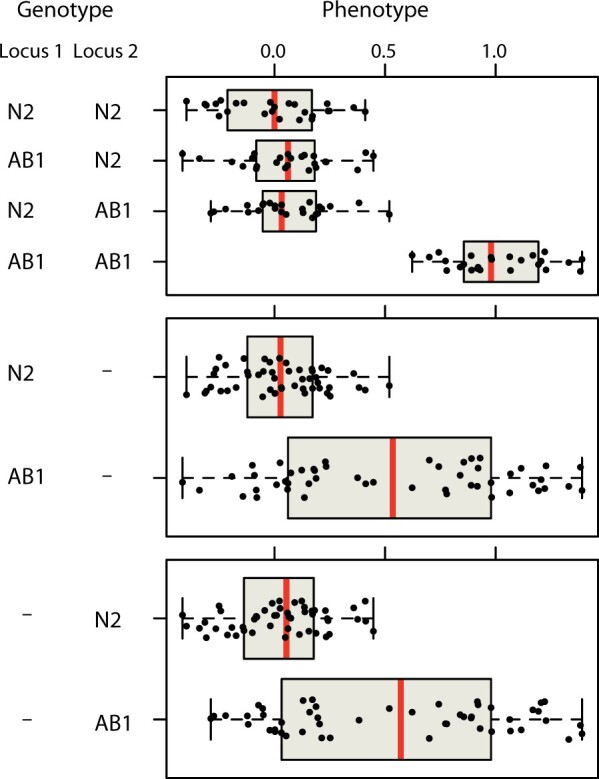
Epistasis for free. A hypothetical example of a trait with pure redundancy between two underlying loci that differ between the strains N2 and AB1. Of 100 simulated RILs, only those carrying AB1 alleles at both loci show a phenotypic effect of the loci (top panel). But considering the loci one at a time (middle and bottom panels), each locus has a clear marginal effect and is easily detectable by QTL mapping methods that do not explicitly consider epistatic interactions. Note that the power to detect these loci is strongly influenced by the frequency of the rare phenotypic class (here, ¼, but often much lower in GWA mapping designs). Methods tailored to discover epistatic interactions often take advantage of the difference in phenotypic variance between single-locus phenotypic distributions to identify candidate interactors (Struchalin *et al.* 2010; Rönnegård and Valdar 2012).

Second, the multifactorial character of quantitative genetics facilitates the efficient discovery of genes whose effects depend on other genetic perturbations, including all of the classic kinds of epistasis, suppressors, and enhancers that can be identified using sequential classical screens, but also pure redundancy, where neither of two single mutations has any effect. This feature of quantitative genetics is not obvious and has been widely misunderstood. The key underlying fact is that multifactorial perturbation generates multiple combinations of alleles across loci. Consider again our hypothetical example of 100 strains, each carrying a uniquely shuffled genome derived from two inbred lines. If a trait is affected by two totally redundant genes, such that only a double mutant shows a mutant phenotype, we expect to observe the rare combination 25 times in our sample, versus rarely in a one-perturbation-at-a-time approach (Ferguson and Horvitz 1989; Andersen *et al.* 2008). Moreover, we will detect these interacting loci for free, without tailoring the analysis to them in any way, because the average effect of each of the two loci is detectable (Figure 2). In *C. elegans*, quantitative genetics pointed to roles for *glb-5 and npr-1* in response to shifts in the ratio of O_2_ and CO_2_, with the effects largely visible only in one of the four two-locus genotypes (see Figure 1 in McGrath *et al.* 2009).

Third, the standard N2 strain we use for *C. elegans* genetics is just a single example of worm biology (Sterken *et al.* 2015), and genes that are missing or broken in this reference strain are invisible to classical genetic analyses. Quantitative genetics has allowed for the discovery of multiple genes that are pseudogenized in the N2 strain [*e.g*., *plg-1* (Palopoli *et al.* 2008), *col-182* (Noble *et al.* 2020), and *glb-5* (McGrath *et al.* 2009)], additional genes where N2 carries hypomorphs [*e.g., nath-10* (Duveau and Félix 2012)], and still other genes that interact with gain-of-function mutations that arose during N2 domestication [*e.g*., *nurf-1* (Xu *et al.* 2019) and *npr-1* (McGrath *et al.* 2009; Andersen *et al.* 2014)]. Moreover, some genes have functions that only make sense in the context of natural genetic variation. For example, *zeel-1 and pha-1* are essential genes in N2; mutations in these genes cause embryonic lethality, but these genes are totally dispensable in other strain backgrounds. They are only essential in N2 because they counteract toxins that are genetically encoded by tightly linked genes that are present in N2 and absent in many other strains (Seidel *et al.* 2008; Ben-David *et al.* 2017).

Fourth, natural genetic variation provides a ready alternative for studies that require quantitative perturbations of biological function, dialing a biological activity up or down to test the relative sensitivities of other processes to these quantitative changes. In conventional molecular genetics approaches, this role is filled by an allelic series or a dose-dependent pharmacological intervention. But allelic series are hard to come by, and pharmacology can be a blunt tool. For biological activities that are affected by many genetic loci—which is to say, for most biological activities—a collection of wild isolates or recombinant lines offers a continuous distribution of that activity, and downstream phenotypes can be tested for their sensitivities. Similarly, because many traits vary simultaneously in a multifactorial perturbation experiment, researchers can isolate the quantitative effects of one variable while accounting for quantitative variation in a second. The ability to test for effects in this kind of multivariate setting allows for tests of causal models that are otherwise experimentally challenging (Rockman 2008; Evans *et al.* 2018; Farhadifar *et al.* 2020).

## The foundations of *Caenorhabditis elegans* QTL mapping

*Caenorhabditis* *elegans* quantitative genetics is the simplest version of quantitative genetics. The main reason is that we study fully homozygous inbred lines. Inbred lines allow us to consider only two genotype classes at each locus, removing dominance from consideration in mapping because no heterozygotes are studied, and they allow each trait to be assayed in large numbers of genetically identical individuals under strictly controlled laboratory conditions. The complex calculations required for working with pedigrees or heterogeneous natural environments are absent. Instead, *C. elegans* researchers can simply measure phenotypes in individuals from distinct inbred lines and then attribute variation among lines to genetic variation. Unlike most other animal models, *C. elegans* usually lives in nature as inbred lines, so the phenotypes of these lines reflect natural variation and not pathologies caused by inbreeding depression (Dolgin *et al.* 2007; Gimond *et al.* 2013; Cutter *et al.* 2019). The second advantage of *C. elegans* is its small, tidy genome, relatively devoid of repetitive elements and completely lacking centromeric repeats. Genomic variation is relatively cheap and easy to measure. Third, *C. elegans* has a wonderfully short generation time, allowing for even arbitrarily complex strain constructions in a short time. Finally, *Caenorhabditis* nematodes can be cryopreserved indefinitely, so that genotyped strains can be maintained without accumulating mutations.

The framework of linear modeling provides a useful way to think about the factors contributing to phenotypic variation. Given a collection of measurements, we can partition variation in the measurements in terms of each of the factors that contribute to variation. For example, if we have measurements of individuals from a single strain raised on two different food sources, with replicates of each, we could write the model *y_i_* = *µ + βF_i_ + ε_i_*. Here, the measured phenotype *y* of individual *i* is modeled as the global mean phenotype *µ*, plus a deviation *β* due to the food source *F_i_*, plus a random error, *ε_i_*, that represents effects of the unique microenvironment of individual *i* along with any measurement error. As we add more variables, our linear model grows and potentially includes interactions among variables. If we have individuals from two different strains in the two environments, we could think of the model *y_i_ = µ + β_G_G_i_ + β_F_F_i_ + β_GxF_(GF)_i_* + *ε_i_*, where the model includes an average effect of the strain genotype (*G*), an average effect of the food source (*F*), and an interaction between genotype and food (*GF*), to account for effects of combinations of strain and food that are not captured by their averages. The coefficients *β*, which represent the effect sizes of the variables in the measured population, can be estimated by linear regression, minimizing the sum of the squared residual error *ε* across the whole collection of observations. With *C. elegans* and its relatives, we often measure phenotypes as the average across all of the genetically identical individuals on an assay plate, and so phenotype *y_i_* refers to the plate-level measurement but logic of the model is otherwise unchanged.

For QTL mapping with inbred lines, we assay a large number of strains and include each strain’s genotype at a locus in the linear model, generically of the form *y_i_* = *µ + β_Q_Q_i_* + *ε_i_*, where *Q_i_* is the strain’s genotype at the site being tested for the presence of a QTL. We then compare this QTL model to a null model in which the locus has no effect, so *β_Q_* = 0. The power to detect a QTL in this setting depends on the product of two numbers: the number of inbred lines assayed and the fraction of phenotypic variance that is attributable to the QTL. That researchers control the number of lines assayed is obvious, but they also have considerable control over the fraction of phenotypic variance attributable to the QTL. That fraction is the ratio of variance due to genotype at the QTL, *V_QTL_*, to the total phenotypic variation, *V_P_*, which includes variation due to the QTL as well as a broad range of other factors, most notably environmental variation and genetic effects of other loci. The QTL mapper can take a variety of steps to maximize *V_QTL_* while reducing other contributors to *V_P_*.

## Increasing *V_QTL_*

The phenotypic variance attributable to a QTL, *V_QTL_*, is maximized when the two genotypic classes (*e.g.*, homozygotes for one allele *vs* homozygotes for the other) are at similar frequencies (50:50). Panels of inbred lines derived from a cross between two founder lines, such as RILs, are designed to maximize *V_QTL_* by fixing allele frequencies at 0.5 genome-wide. Conversely, panels of wild isolates sample allele frequencies from nature, and QTL studied in such panels often have uneven frequencies, with one allele much rarer than the other.

*V_QTL_* is more complicated for epistatic interactions. For interactions of the sort shown in Figure 2, three of the four genotypes have the same mean phenotype, and so the genotypic classes that matter are present in frequencies ¼ and ¾. For an interaction that depends on three loci, the rare class would be present in only 1/8 of the lines, if allele frequencies among the inbred lines are 50:50 at each locus. For this reason, the *V_QTL_* caused by epistatic interactions, though it contributes to additive variance as shown in Figure 2, is quite sensitive to allele frequencies, and higher-order epistatic effects contribute so little additive variance as to be nearly invisible. For similar reasons, experiments designed to assay genotype-by-environment interactions should carefully consider the sample size requirements for the specific goals.

*V_QTL_* may be masked by linkage of the QTL to other loci with opposing effects, so recombination is another important tool to increase the variation due to a QTL. A common phenomenon in *C. elegans* quantitative genetics is that RILs derived from wild isolates with similar phenotypes vary far beyond the phenotypic range of the founding strains. This pattern is known as transgressive segregation and is a result of recombination shuffling the alleles into new combinations. If each founding wild isolate carries a collection of trait-increasing and trait-decreasing alleles, the founders may be phenotypically similar, but their recombined descendants could end up with all trait-increasing or all trait-decreasing alleles. Transgressive segregation means that QTL mapping is often successful in *C. elegans* even when the founding strains are phenotypically identical (*e.g*., Farhadifar *et al.* 2020). It may also mean, for highly polygenic traits, that individual QTL with moderate to large effects are actually composed of smaller effect QTL that are tightly linked and evade detection unless special effort is made to break up linked loci (*e.g*., Bernstein *et al.* 2019).

## Decreasing all the other sources of *V_P_*

Power to detect a QTL increase as phenotypic variation from all other sources is reduced. *C. elegans* researchers have many tools to reduce this residual variation.

The ordinary routines of laboratory control are essential for reducing random environmental variation, including controls on temperature, humidity, plate media, food, worm manipulation, and so forth. Because of the prevalence of transgenerational environmental effects in *C. elegans*, researchers should control conditions for several generations prior to assays (Sakaguchi *et al.* 2014; Heestand *et al.* 2018; Webster *et al.* 2018; Moore *et al.* 2019; Baugh and Day 2020; Baugh and Hu 2020; Ewe *et al.* 2020; Houri-Zeevi *et al.* 2020). Careful environmental control is standard operating procedure in every worm lab, but QTL mapping introduces new complications. Regardless of the quantitative genetic mapping method, the phenotypes of many independent strains must be measured accurately. This scaling issue is quite different from what the typical *C. elegans* laboratory encounters, where studies are often focused on the N2 strain and a few mutant strains. Phenotyping methods that can measure traits across minimally tens but oftentimes hundreds of independent strains at one time are preferred in order to reduce environmental and assay-to-assay variation. High-throughput measurements have been successful in measurements of offspring production (Andersen *et al.* 2014, 2015), growth rate (Cook *et al.* 2016a; Zdraljevic *et al.* 2017; Hahnel *et al.* 2018), and behaviors (McGrath *et al.* 2009; Reddy *et al.* 2009; Bendesky *et al.* 2012; Ghosh *et al.* 2012a, 2012b, 2015).

In most cases, the scale required precludes simultaneous assays on all strains. When subsets of strains are measured on different days, environmental factors that vary day to day contribute to *V_P_*. These batch effects represent systematic environmental perturbations, like the food source in our example linear model, and unlike the random microenvironmental variation that makes genetically identical individuals different from one another on a single plate. For QTL mapping, batch effects are often handled by explicitly including batch identity in the linear model used for mapping. A similar approach is to adjust phenotype values prior to mapping, by regressing out the batch effects. Ideally, each inbred line will be included in multiple batches, and the collection of lines will be randomized across batches. Randomization is good practice generally, but in particular researchers should avoid batching strains according to trait values. For example, measuring fast-growing lines one day and slow-growing lines the next day introduces a confounding between batch effects and genotypes. This is a frequent concern in agricultural and medical genetics, where this kind of genotype-environment covariance (*Cov_G, E_*, not to be confused with GxE, or genotype-by-environment interaction) is common, but *C. elegans* researchers can simply avoid it with careful experimental design.

A simple way to increase *V_QTL_/V_P_* is to reduce noise in the phenotype measurements, which can be done on a per-worm basis, for example by taking multiple measures of each worm, or tracking a worm’s behavior traits over longer time periods. Importantly, inbred lines provide an even better option to measure multiple worms per line. For QTL mapping with inbred lines, the phenotypic variation of interest is the among-line variance, not the among-worm. Consequently, the better each line’s mean phenotype is measured, the more the total variance will reflect genetic effects. Notwithstanding this benefit from measuring many worms per line, an investigator with the choice to increase the number of worms per line or to increase the number of lines will almost always be better served by increasing the number of lines. Indeed, because QTL mapping leverages randomized multifactorial perturbation, replicate measurements of each line are really not required. An additional caveat is that phenotypes measured on multiple genetically identical worms on a single plate represent a single measurement because worms share a common environment; that is, in estimating a strain’s phenotype, three plates of 100 worms each is closer to three measurements than to 300. Researchers typically take the mean of each plate as one measurement and consider *V_P_* as the among-plate variation, partitioned into within-line and among-line.

With the mapping goal of maximizing *V_QTL_/V_P_* in mind, researchers can experiment with a range of environmental conditions, assays, or phenotype index (*e.g*., a weighted combination of phenotypes, though this may reduce interpretability), in order to find an experimental setup that has high among-line variance. A QTL that contributes no variance at 20°C on OP50 could have a large effect at 18°C on HB101. In the case of cryptic genetic variation, which generates *V_QTL_* only under rare conditions, more extreme perturbations are required (Paaby *et al.* 2015; Vu *et al.* 2015; Torres Cleuren *et al.* 2019).

Finally, researchers may want to account for genetic factors other than the focal locus that contribute to *V_P_*. That is, to test for an effect of one region of the genome, phenotypic variation due to genetic variation elsewhere in the genome may reduce detection power by increasing *V_P_* and thereby decreasing the fraction of variance due to our test region. When the background QTL are known, they can be accounted for much like batch effects, by including them in the linear model or regressing them out in advance. They can also be experimentally removed from consideration, by generating new mapping panels in which the known variants are fixed (*e.g*., Sinha *et al.* 2008; Andersen *et al.* 2015). Accounting for background genetic variation is particularly important in GWA mapping, where its complex structure can otherwise contribute misleading signals, and powerful statistical approaches (discussed in the GWA section below) provide the necessary accounting.

## Genetic variation in *Caenorhabditis elegans* and other *Caenorhabditis* species

QTL mapping makes use of naturally occurring genetic variation as a source of function-perturbing mutations. The set of variants that go into an experimental mapping panel sets bounds on the scope of genetic perturbations we can study, influences the ease with which we can localize causal variants, and defines the range of evolutionary insights we can draw. For example, consider an experiment that maps QTL in a cross of two closely related strains. Closely related strains differ at few sites, so the probability that they differ at a QTL is low; few variants means few molecular processes perturbed. At the same time, mutations that differentiate closely related strains likely have arisen recently, and so are likely rare in the species as a whole. That also means that they have been relatively untested by natural selection in the wild, and they may therefore have larger effect sizes than more ancient variation. Finally, because there are few variants, the challenge of picking out causal variants within a QTL will be relatively easy, as there will be fewer candidate genes harboring variation. Alternatively, crosses between divergent strains provide greater assurance that QTL will be present, but they also increase the challenges of identifying causal variants from the large number of variants that will occur within each QTL region. Divergent crosses may also increase the probability of outbreeding depression, where alleles that have coevolved in separate lineages interact badly when brought together in recombinant mapping strains. The mean fitness in a panel of lines from a divergent cross may be lower than the fitnesses of the parental strains, and so measurements of specific phenotypes may reflect generic pathology rather than features specific to the focal trait. Crosses between two strains provide a narrow view of genetic variation in a species, and mapping in panels of wild isolates (*i.e*., GWA studies) typically provide a broader perspective. This wide view trades off with the different, and generally lower, statistical power for QTL detection in GWA mapping.

In *C. elegans*, a study of 609 wild strains, nearly the complete catalog of known isolates, identified 2,431,645 single-nucleotide variants and 845,797 small (≤50bp) insertions and deletions (Lee *et al.* 2021). In addition, the study showed that the species harbors hundreds of regions where hyperdivergent haplotypes segregate. Haplotypes are groups of alleles that are inherited as a unit from a single parent because they occur together on a segment of chromosome. Determining the total number of variants within these regions awaits species-wide long-read sequencing. In the meantime, we can confidently say that more than 3% of all sites in the ∼100 Mb reference genome vary in the *C. elegans* population and 89% of all N2 genes have a predicted deleterious variant in at least one wild *C. elegans* strain, providing an enormous pool of genetic perturbations.

Comparable species-wide data will soon be available for *C. briggsae and C. tropicalis*. In the meantime, a survey of 37 *C. briggsae* isolates identified more than three million SNVs and small indels (Thomas *et al.* 2015), and a study of 24 isolates of *C. tropicalis* identified fewer than a million SNVs (Noble *et al.* 2021b). Assuming those studies surveyed a representative sample of strains, we can conclude that *C. elegans* is intermediate among the selfers in its level of genetic variation. Like *C. elegans*, both *C. briggsae* and *C. tropicalis* also carry extensive regions of hyperdivergent haplotypes whose contributions to coding sequence variation remain to be fully characterized (Lee *et al.* 2021; Noble *et al.* 2021b).

The dozens of other *Caenorhabditis* species are all gonochoristic, with separate males and females and obligate outcrossing every generation (Kiontke *et al.* 2011; Félix *et al.* 2014; Yin *et al.* 2018; Stevens *et al.* 2019, 2020; Dayi *et al.* 2021). Many of these species harbor enormous quantities of genetic variation; *C. brenneri* may be the most genetically diverse of all animal species (Dey *et al.* 2013). The genetic variation in these outcrossers includes a large store of recessive deleterious variation that renders them nearly incapable of inbreeding to homozygosity (Dolgin *et al.* 2007; Barrière *et al.* 2009). Substantial efforts have generated some nearly homozygous strains and relatively high-quality reference genomes for *C. remanei* (Fierst *et al.* 2015; Teterina *et al.* 2020), which is currently the preeminent gonochoristic *Caenorhabditis* species for quantitative genetic studies (Reynolds and Phillips 2013; O’Connor *et al.* 2021). Some gonochoristic species appear to have more modest levels of genetic variation, and these species might be more amenable to quantitative genetic methods that rely on inbred lines (Li *et al.* 2014; Stevens *et al.* 2020). For all of the gonochoristic species, however, incomplete and poorly validated gene models remain an obstacle to functional discovery.

## Quantitative genetic mapping requires determination of genotype

One of the most powerful advantages of *C. elegans* quantitative genetics is that mapping panels and strain resources can be constructed (or collected), genotyped once, cryopreserved, and then phenotyped in any lab. This process is dependent on identification of the genotypes of these strains. The advent of inexpensive whole-genome sequencing has made this process straightforward. Many strains can be multiplexed and sequenced in the same run, reducing costs. This point is especially clear when genotyping inbred strains that are derived from crosses of well-characterized founders (RILs, RIAILs, NILs, MPPs, and BSA; Figure 1). In these cases, low-coverage sequencing (approximately 1x) of each strain is sufficient to impute genome-wide genotypes with high confidence (Noble *et al.* 2017; Evans *et al.* 2018a, 2018b; Na *et al.* 2020). Whole-genome resequencing of wild isolates requires higher coverage but also scales well for *Caenorhabditis* species that have small genomes.

All genotyping endeavors are influenced by two important points. First, they are biased by the reference genome used for short-read sequence alignments. If the strain has a genome quite different from the reference genome, then some reads will not align, variants will not be identified in those regions, and genotypes will not be determined for the entire genome. This point is most easily observed in the case of hyper-divergent regions (Lee *et al.* 2021; Noble *et al.* 2021b) where genome regions are completely different from the reference strain and contain tens to hundreds of new genes and undiscovered biology. Second, genotyping is also affected by the types and positions of variants that can be called using different sequencing technologies. The Illumina short-read sequence platform enables scaling of highly multiplexed sequencing but it can not identify variants well that are longer than the read length. For this reason, variants that cause phenotypic differences across a population might not be in the whole-genome genotype data or even mapping marker set. New long-read sequencing technologies can be used to address the other classes of variation, but algorithms and methods are still actively being developed. Importantly, these concerns are typically irrelevant for mapping QTL. As long as the undetected variants are correlated with observed variants, associations between the undetected variants and phenotypes will be detected using their shared correlations with the observed variants. This logic is fundamental to genetic mapping, including GWA in humans: LD makes observed variants informative about unobserved variants. The length-scale of LD in *C. elegans—*whether in wild isolates or experimentally generated lines—is sufficiently long that linkage and GWA mapping work well for detecting genomic regions containing QTL, even with imperfect genotyping. However, when attempting to pin down the precise variants responsible for phenotypic variation, in the fine-mapping stage of a QTL mapping project, unobserved variants can lead to erroneous inferences. Overall, genotyping *Caenorhabditis* strains is facilitated by small genomes, defined chromosomal domains, and a rich history of comparative genomics.

## Experimental designs for mapping

### Linkage mapping—correlating genotype and phenotype using recombinant lines

As we explained above, quantitative genetic mapping correlates genotype and phenotype to identify QTL. Tests of correlation are most powerful when the population is approximately evenly split between two genotypes. To create a population with approximately equal allele frequencies, researchers generate panels of recombinant inbred lines (RILs), each derived by reshuffling the genetic differences between two founding inbred lines (Figure 1). In the absence of selection, the final panel will have approximately equal contributions from both strain genetic backgrounds throughout the genome. RILs carry pieces of their founding genomes in relatively large segments, so beyond the advantages in allele frequencies, linkage mapping in RILs can also leverage sparse genotyping and perform fewer statistical tests of correlation across the genome. These analyses generate confidence intervals where causal quantitative trait genes (QTGs) likely reside. RIL panels are constructed and genotyped once but can be phenotyped repeatedly for many traits. Combined with cryopreservation, RIL panels are living resources usable in perpetuity.

**Figure 3:**
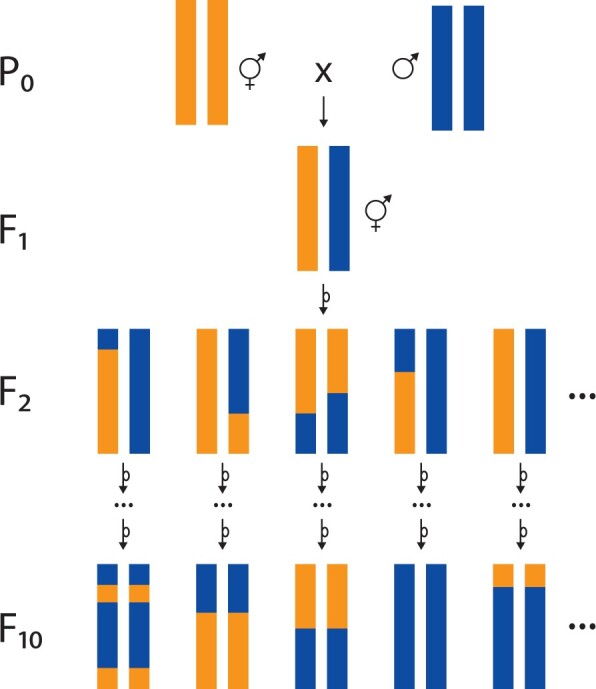
RIL construction. Conventional RILs are generated by crossing two inbred lines (P_0_), each here represented by a single pair of homologous chromosomes. The cross yields F_1_s that are heterozygous at every locus that differs between the strains. Self-fertilization of F_1_ hermaphrodites yields genetically heterogeneous F_2_s. Because of complete crossover interference in *C. elegans*, each F_2_ carries on average one recombinant chromosome with a single crossover. Each F_2_ is independently inbred by selfing until the F_10_ generation, at which point the genome is expected to be completely homozygous, with an average of one crossover per chromosome. This collection of homozygous strains is a panel of RILs. Note that this panel will all inherit the mitochondrial genome of the P_0_ hermaphrodite. To balance the panel with respect to mitochondrial genotype, an equal number of RILs should be constructed from the reciprocal cross.

*C. elegans* usually has only one crossover per chromosome per meiosis (Hillers and Villeneuve 2003), limiting the degree of randomization among alleles. Conventional RILs are generated by inbreeding from a simple F_2_ population. The resulting RILs have few recombination breakpoints but are easy to construct (Figure 3). With few breakpoints comes low mapping resolution—each variant will be highly correlated with neighboring variants across a long segment of chromosome. To ameliorate this problem, an alternative experimental design, recombinant inbred advanced intercross lines (RIAILs), is widely used. RIAILs are generated by many generations of crossing of recombinant individuals to one another, so they have many recombination breakpoints. RIAILs are more labor-intensive than RILs to construct and genotype but the effort is justified by greater mapping resolution.

The construction of these panels is divided into two phases: crossing and inbreeding. Both RILs and RIAILs start the crossing phase with a cross of two genetically (and oftentimes phenotypically) divergent strains. To balance mitochondrial genotypes, pairwise crosses with males and females of each of the two strains should be performed (strain A male × strain B hermaphrodite and strain B male × strain A hermaphrodite). Four types of F_1_ individuals from these two crosses will be generated: males or hermaphrodites with either strain A or B mitochondria. For RILs, the two hermaphrodite classes can be selfed. For RIAILs, the F_1_ individuals can be crossed in each of four combinations to mix different mitochondrial and X-chromosome genetic backgrounds. Recombination in the F_1_ parents will create unique combinations of the two genetic backgrounds (A and B). The F_2_ individuals will harbor a collection of different recombinant and parental chromosomes. RIAIL panel construction continues the crossing phase by intercrossing the F_2_ (and subsequent recombinant generations) to each other (Rockman and Kruglyak 2008). The RIAIL intercrossing phase produces a collection of recombinant individuals with unique breakpoints throughout the genome, but these individuals are heterozygous at many loci. These individuals must be homozygosed so that cryopreservation will maintain stable genetic backgrounds. To homozygose recombinant genotypes, line construction projects enter the inbreeding phase. Individuals from the RIAIL crossing phase or F_2_ individuals from the RIL crossing phase are selected and propagated by single-hermaphrodite passage for ten generations, enough so that each locus that differed between the founding lines as a low probability (1/2^10^) of retaining heterozygosity. For obligate outcrossers, inbreeding is achieved by sib-mating, which takes more generations to reach the same degree of homozygosity; researchers typically aim for 20 generations of sib-mating, which gives a locus a 99% probability of homozygosity, absent selection (*e.g*., Wright 1921). The final size of a panel is dependent on the number of unique recombinant individuals selected for the inbreeding phase and the loss of lines during the inbreeding phase.

After genotyping the inbred lines, allele frequency skews are sometimes observed in genomic locations where incompatibility loci are found (Seidel *et al.* 2008, 2011; Ben-David *et al.* 2017; Ross *et al*. 2011; Noble *et al.* 2021b). The known large-effect incompatibilities all involve a special class of single-locus parent-offspring interaction that only manifests in the progeny of heterozygotes. Consequently, these loci contribute to allele frequency distortion during line construction but not to phenotypic variation among inbred lines. At the same time, selfing *Caenorhabditis* species also exhibit outbreeding depression due in part to weakly incompatible alleles (Snoek *et al.* 2014). These interacting loci are common and cause subtle effects on fitness. Generally speaking, allele frequency skews reduce statistical power to detect QTL in the region of the skew, but they pose little risk for creating false positives.

Linkage mapping in *C. elegans* leverages recombinant line panels to measure phenotypic variation and correlate with genotypic variation to identify QTL. With an abundance of genetic markers spread evenly throughout the genome, a marker-based regression approach is often performed for linkage mapping. Much of the traditional statistical opacity of QTL mapping is due to complications of sparse marker data, a problem that genome sequencing has largely solved. For each marker throughout the genome, the RIL collection is divided into the two founder genotypes. Then, a linear model is generated to describe the difference in phenotype between these two groups. This linear model is compared to a model in which the recombinant panel is not divided into two groups based on the founder genotype. These calculations facilitate the comparison of models by constructing an odds ratio between the goodness of fit of both models. The LOD can be calculated to determine how different these two models are. A LOD score of three indicates that the data are 1000 times more probable under the linear model with two groups than under the model with one group, a good correlation of genotype with phenotype at this marker.

**Figure 4:**
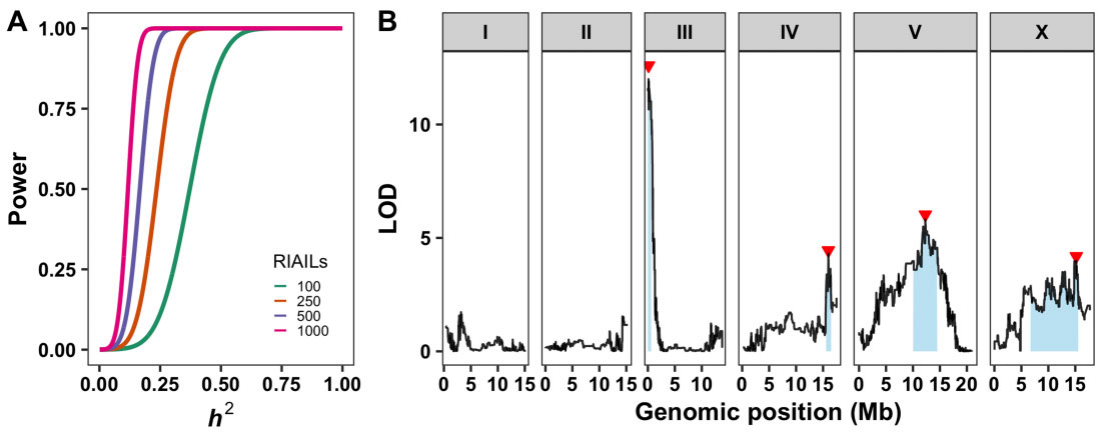
Linkage mapping power and example. (A) The statistical power of different RIAIL panel sizes is plotted by the QTL effect (percent of *V_P_* that is *V_QTL_*). Larger RIAIL panels can identify QTL that in total explain more of the total phenotypic variance. These data were generated using the QTLDesign package in R (Sen *et al.* 2007). (B) An example linkage mapping plot for response to zinc (Evans *et al.* 2020) is shown. Genomic position (*x*-axis) is plotted against the logarithm of the odds (LOD) score (*y*-axis) for 13,003 genomic markers. Each significant QTL is indicated by a red triangle at the peak marker, and a blue rectangle shows the 95% confidence interval around the peak marker.

The statistical model that allows the two genotype groups to have different mean phenotypes will always fit better than the null model in which the groups share a single mean, and consequently LOD scores of this sort are always positive. Further, because the statistic is calculated for each marker throughout the genome, strikingly high LOD scores will occur at some markers by chance, even if those regions do not have QTL (*e.g.*, see the peak on chromosome I in Figure 4B). To determine which genomic positions are significantly correlated with phenotypic differences, a permutation-based approach is used (Churchill and Doerge 1994). The recombinant line identity is shuffled to break correlations between genotype and phenotype. Then, the genome-wide marker-based regression approach is repeated, yielding LOD scores in the absence of real QTL effects. After hundreds or thousands of rounds of shuffling and calculating scores, the LOD score corresponding to a genome-wide error rate of 5% can be determined by taking the 95th percentile LOD score from the distribution of the maximum LOD score from all the permutations. This 95th percentile represents the LOD score that we would expect to match or exceed somewhere in the genome one time in 20, in the absence of any true QTL (*i.e*., genome-wide *p* = 0.05, *e.g.*, see peaks labeled with red triangles in Figure 4B). This threshold depends somewhat on idiosyncrasies of the phenotype distribution, but mostly on the number of statistically independent regions of the genome: the number of chromosomes and the genetic length of each (Lander and Botstein 1989). More independent regions means more chances for a high LOD score by chance, and so longer genetic maps (*e.g*., RIAILs *vs* RILs) end up with higher thresholds for significance. This relationship creates a trade-off between QTL mapping resolution (improved by a long map) and QTL detection power (reduced by a long map). Typically, a forward-search mapping approach is performed where the most significant QTL position from one round of mapping is used as a covariate in the next round of mapping (Brady *et al.* 2019). This process is repeated until no more significant QTL are detected. This approach enables detection of small-effect QTL and also mitigates concerns about QTL that might be dependent on multiple genomic regions, as is often the case in complex traits.

To define the regions where the causal variant(s) could lie, 95% confidence intervals surrounding the peak QTL marker are calculated. A 1.5 drop in the LOD score from the peak marker closely approximates this level of statistical confidence (Broman and Sen 2009), so it is often reported. It is important to consider that the causal variant(s) might not lie in the QTL confidence intervals. The LOD score is influenced by the allele frequency of the marker and variation contributed by other factors independent of the tested marker. As the allele frequency departs from 50:50, the linear model fit is more affected by outliers. The number of RILs influences the power to detect QTL and the accuracy of those QTL positions. It is best to score as many lines as can be scored in any trait of interest to increase statistical power to detect QTL (Figure 4A). QTL that explain more than 10% of the phenotypic variance can be more easily followed-up using near-isogenic line (NIL) and candidate gene approaches discussed below.

Typically, researchers apply a conventional linear model to calculate linkage statistics. This model treats the data as drawn from normal distributions whose means differ as a function of genotype. Many phenotypes have different distributions, but the linear model approach is versatile and can accommodate traits that are binary, mixtures of binary and continuous, or idiosyncratic in other ways. For example, discrete traits with genetically variable penetrance yield presence/absence data for individual worms, and these data can be analyzed using logistic regression (*e.g*., Noble *et al.* 2015). Nonparametric linkage mapping uses the ranks of the phenotypes rather than their actual values, removing distributional assumptions, albeit at a potential cost in terms of statistical power (*e.g*., Reddy *et al.* 2009; Rockman *et al.* 2010). In general, any statistical test that assesses differences between groups can be adapted to linkage mapping. The Andersen lab created an R package for *C. elegans* and *C. briggsae* linkage mapping using many of the existing RIL and RIAIL strain sets (https://github.com/AndersenLab/linkagemapping).

The linkage mapping approach identifies QTL that harbor allelic differences between two founder strains. Remember that, because of transgressive segregation, it is still possible to detect QTL even if the two founder strains do not have different phenotypes. A linkage mapping approach is designed primarily to detect QTL that contribute additively to the trait variance, meaning that, regardless of phenotypic direction of effect, the founder strain difference is the simple sum of each of the QTL effects taken independently. Many traits are controlled by interacting loci, where the effects of each QTL are enhanced or suppressed by the effects of other loci (Phillips 2008). In rare cases, interaction effects precisely cancel out additive effects (symmetric sign epistasis), and two-factor linkage mapping approaches (*e.g.*, Sen and Churchill 2001; Gaertner *et al.* 2012) are required to detect these loci. However, interactions typically generate additive effects that are visible to conventional linkage mapping (Figure 2), and most (but not all) traits have a primarily additive genetic basis (Bloom *et al.* 2013, 2015; Zdraljevic *et al.* 2017, 2019; Evans *et al.* 2018, 2020).

After a QTL is detected, plots of the phenotype values of all recombinant lines by the founder genotype difference at the peak QTL marker help to visualize the magnitude and direction of the QTL effect. The QTL with the largest phenotypic effects will be easier to narrow to candidate genes and test using candidate gene approaches as described below, but these QTL might not represent evolutionarily important loci where effects might be more modest (Rockman 2012).

So far, we have presented linkage mapping approaches using two founding strains that are from the same species and not genetically modified. Linkage mapping can also be used to identify QTL between different species (*e.g*., Woodruff *et al.* 2010) or QTL that might modify mutant phenotypes (Duveau and Félix 2012; Schmid *et al.* 2015; Koneru *et al.* 2021). If interspecies crosses produce viable offspring, then recombinant lines can be generated between different species. The possibility of hybrid incompatibilities increases as the genetic distance between the two species increases, so allele frequency skews might be more common. For this reason, the construction of recombinant lines is often difficult, so a RIL panel might be preferred over a RIAIL panel. To identify modifiers present in diverse genetic backgrounds, linkage mapping can be applied to founding strains with edited or altered genomes. For example, Kammenga and Felix labs sought to identify modifiers of the RTK/Ras pathway present in wild *C. elegans* strains so they introgressed mutations that cause incompletely penetrant vulval defects in the N2 strain background into wild strain backgrounds (Duveau and Félix 2012; Schmid *et al.* 2015). They each created RIL panels and then measured the modification of the vulval development phenotype in these RILs. RIL panels can also carry mutations that make phenotyping easier, as in the case of a panel that carries a *him-5* mutation, increasing male frequency, to facilitate scoring male traits (Noble *et al.* 2015). In other cases, panels include fluorescent reporters that simplify cellular phenotyping (Koneru *et al.* 2021). These approaches are made significantly easier using the CRISPR-Cas9 genome-editing system to add specific mutations or variants to defined genetic backgrounds for new recombinant line panels. The linkage mapping approach is most powerful in the three selfing *Caenorhabditis* species because genetic diversity is lower and inbreeding is easier than in outcrossing species. However, linkage mapping can work well in outcrossing species when strains can be established with reduced diversity and heterozygosity so that recombinant lines can be crossed and inbred. Overall, the linkage mapping approach using inbred lines is the quantitative genetic mapping method with the greatest power for QTL detection.

*C. elegans* linkage mapping experiments using the N2 and CB4856 strains have been used extensively (Rockman and Kruglyak 2009; Andersen *et al.* 2015; Brady *et al.* 2019). These lines are available from the *C. elegans* Natural Diversity Resource (Cook *et al.* 2016b). For many traits, a few large-effect QTL have been identified. Other traits are affected by large numbers of small-effect loci. In a recent study, four QTL distributed on different chromosomes were found to contribute to exogenous zinc responses (Evans *et al.* 2020) (Figure 4B). In three of the four loci, the N2 allele caused zinc resistance, and the other QTL had the opposite effect with the CB4856 allele causing zinc resistance. One of the QTL was narrowed to a genomic interval that contained a small number of candidate genes on chromosome III and then the candidate gene (*sqst-5*) was shown to mediate the difference in zinc response. Genome-editing approaches (discussed below) were used to show a definitive connection between *sqst-5* variation and differences in zinc responses.

A large number of traits have been studied using panels of N2xCB4856 RIAILs and RILs, which have led to the identification of many genes and specific variants that contribute to natural variation across the *C. elegans* species. Prior to the introduction of the N2xCB4856 recombinant panels, other wild strains were used. Most notably, the Bergerac strain, which goes by numerous strain designations, was crossed to N2 and facilitated early QTL mapping in the species (reviewed in Gaertner and Phillips 2010).

Whole-genome assemblies for the N2 and CB4856 strains identified 327,050 SNVs and 79,529 insertions and deletions, as well as 816 segments too divergent to align (Thompson *et al.* 2015). Some 8,140 protein-coding genes (40%) harbor protein-altering variants, including 1,885 with apparent loss-of-function mutations. In addition, thousands of genes show evidence of *cis*-acting regulatory differences between these strains (Li *et al.* 2006; Capra *et al.* 2008; Rockman *et al.* 2010; Viñuela *et al.* 2010; Andersen *et al.* 2014; Francesconi and Lehner 2014). Overall, inbred line panels derived from these strains provide an efficient way of assaying the effects of perturbations to a large number of genes and molecular processes. At the same time, fine mapping is necessarily limited by the relatively large number of variants compared to the number of recombination events that separate them during RIL or RIAIL construction.

Researchers have also studied traits in *C. briggsae and C. tropicalis* using RIL panels. In *C. briggsae*, RILs and RIAILs derived from the Indian strain AF16 and the Japanese isolate HK104 have revealed genetic factors contributing to variation in male tail ray development and drug sensitivity (Baird *et al.* 2005; Ross *et al.* 2011; Zamanian *et al.* 2018). In *C. tropicalis*, RILs derived from strain NIC58 from French Guiana and strain JU1373 from Reunion Island have been used to map a QTL affecting hermaphrodite mating propensity and genetic incompatibilities (Noble *et al.* 2021b).

### Genome-wide association mapping—correlating genotype and phenotype using wild strains

Natural populations harbor staggering levels of genotypic and phenotypic diversity. Heritable phenotypic diversity is the product of mutation, selection, and drift, so the underlying genotypic diversity reflects those causes. Unlike linkage mapping where genetic differences between two strains are tested for correlations with phenotypic differences, GWA mapping leverages the diversity and historical recombination found across populations to identify loci that could underlie phenotypic variation in those populations. The number of variants tested for correlations is typically much higher, the allele frequencies of those variants depart significantly from 50:50, and correlations among variants (LD) cause more unpredictable effects on GWA than linkage. In this section, we will describe the process, power, and caveats of GWA mapping in *C. elegans* and related *Caenorhabditis* nematodes.

**Figure 5:**
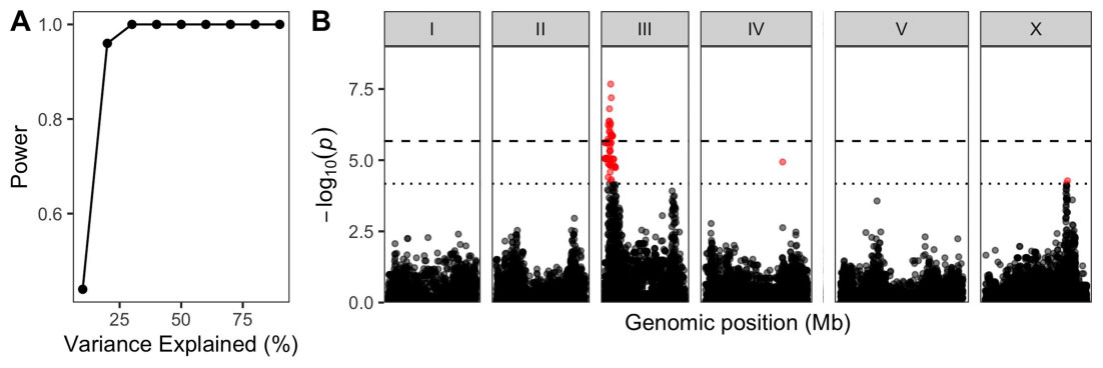
Genome-wide association mapping power and example. (A) Statistical power for the 20200815 CeNDR release strain set is plotted by the QTL effect (Percent of phenotypic variance explained by the QTL). (B) A Manhattan plot for single-marker-based GWA mapping of the ascr#5-induced dauer formation trait (Lee *et al.* 2019) is shown. Each dot represents a single-nucleotide variant (SNV) that is present in at least 5% of 157 wild strains. The genomic position in Mb, separated by chromosome, is plotted on the *x*-axis, and the statistical significance of the correlation between genotype and phenotype is plotted on the *y*-axis. Two significance thresholds are shown. The dashed horizontal line denotes the Bonferroni-corrected *P*-value threshold using all markers, and the solid horizontal line denotes the Eigen-corrected *P*-value threshold using independent markers correcting for LD (genome-wide eigen-decomposition significance threshold). SNVs are colored red if they pass either threshold.

GWA mapping correlates phenotypic variation among wild strains with whole-genome variant data to identify QTL. In *C. elegans*, a growing collection of wild strains and whole-genome sequence data are available in the *C. elegans* natural diversity resource (CeNDR). Wild strains are grouped into isotypes where the strains within an isotype are highly related to each other. If all wild strains are used in mapping, then these highly related strains will bias statistical tests while adding little additional information. For this reason, CeNDR recommends using isotype reference strains (the chosen reference for each isotype) in all studies of wild strains. These isotype reference strains are organized into sets of 48 to facilitate GWA mapping experiments. Statistical power calculations show that a minimum of 96 strains should be scored to have 80% power to detect QTL that explain 15% of the variation in that population (Figure 5A). In addition to performing a GWA mapping, the phenotypes of wild strains can be used to determine founding strains for additional RIL collections and/or bulk-segregant mapping approaches.

On the genotype side, CeNDR provides releases of whole-genome sequence and variant data for all strains. For GWA mapping, one does not typically test every variant in the genome for a correlation with phenotypic variation. First, many variants are rare (allele frequencies less than 5%), which reduces power to detect QTL if they were included in the GWA mapping. Second, alleles at distinct loci can be correlated (LD is high in selfing species like *C. elegans*), rendering them redundant. Whole-genome variant data are pruned to reduce these effects. In the CeNDR GWA workflow, bi-allelic variant sites with more than 10% missing variant calls across the population are removed. The remaining variant sites with minor-allele frequencies less than 5% are removed. Last, LD between all remaining variant sites is assessed and pairs of variant sites that are correlated greater than 0.8 are pruned to just one of the two sites. These steps typically reduce the species-wide variant data set from millions to tens of thousands of variant sites.

Some regions of the genome have relatively few variants for three different reasons. First, centers of chromosomes, where recombination is relatively low, harbor fewer variants than chromosome arms because of the effects of background selection (Cutter and Payseur 2003; Rockman *et al.* 2010). Under background selection, the constant influx of new deleterious mutations reduces genetic diversity at sites linked to the mutations. Second, selective sweeps have removed diversity on chromosomes I, IV, V, and X (Andersen *et al.* 2012). A selective sweep results when a new beneficial mutation spreads through the population quickly, replacing the diverse chromosomes that do not carry the mutation. Third, variant calling is more difficult, if not impossible, in divergent regions where the wild isolate genome is extremely different from the N2 reference genome and short-read sequences do not align to the reference genome (Thompson *et al.* 2015; Lee *et al.* 2021).

Current GWA mapping methods use a linear mixed-effect model approach to identify genotype-phenotype correlations (see Sul *et al.* 2018 for a good explanation). In this approach, the genetic variant being tested for an effect on phenotype is modeled exactly as in the linear models described previously. The genotype at the locus has a fixed effect whose sign and magnitude we hope to estimate. The other part of the mixed-effect model is the random effect, where we model variation in phenotype caused by nuisance variables, factors that we have to account for even though their exact values are not our interest. Rather than estimate their values, we treat them as random—that is, as values drawn from a probability distribution—and we attempt to model the shape of that distribution. In GWA mapping, random effects are included to account for spurious correlations that are caused by related strains within the mapping population. Related individuals are often found in nearby areas. This situation is called population structure and can be described by a matrix of pairwise sharing of genome-wide variants among the strains in a population (Sul *et al.* 2018). Without adjustments for population structure, every genomic region that is genetically similar among strains that are phenotypically similar will look like a QTL, even though many such regions will simply reflect the shared history of the related strains. As a random effect in the mixed model, the population structure matrix effectively down-weights observed phenotypic similarities among strains that are also genotypically similar genome-wide. In some cases, a QTL effect might be correlated with the population structure, which will decrease the likelihood that a QTL will be detected. Because of the differences in distribution of variants across and within chromosomes, statistical power to detect QTL is higher on chromosome arms where more intermediate and common allele frequency variants are found. In addition, LD is less on chromosome arms so mapping resolution is higher.

As in the case of linkage mapping, a large number of statistical tests will result in a large number of associations, even in the absence of QTL. To determine which of the many associations between genotype and phenotype are statistically significant, the significance threshold (alpha, conventionally 0.05) is adjusted for the number of independent tests. The most conservative significance threshold uses the Bonferroni method, where alpha is divided by the number of markers where the test of association was performed. This method is designed to establish the probability that a QTL is detected anywhere in the genome at 5%, under the null hypothesis that no QTL exist. However, Bonferroni is overly conservative because many of those markers are not independent because of LD; that is, Bonferroni divides alpha by an inflated number in this context, setting the threshold for significance at an inappropriately stringent level. Alternatively, alpha can be adjusted by dividing it by the effective number of independent markers across the genome. The variant pruning steps explained above reduce many dependent markers but not all. To determine the effective number of independent markers, the correlation among markers in the genotype matrix can be reduced by Eigen decomposition (Li and Ji 2005). The experimentalist can decide whether to focus on QTL defined by the conservative Bonferroni threshold or the more liberal Eigen-value threshold. QTL detected using either threshold have been validated using NILs and genome-edited strains.

Permutation approaches, such as those used in linkage mapping, are generally not appropriate for GWA. The logic of permutation is that the shuffled strains are interchangeable under the null hypothesis of no QTL (Churchill and Doerge 1994). With wild isolates, however, the strains are not interchangeable, because some are very similar to one another and others very different (Churchill and Doerge 2008; Abney 2015). As mixed-model approaches have become more popular, permutations have been adapted to significance testing in the GWA mapping context. For example, if phenotypes are adjusted by regressing out population structure effects, the significance of associations between a locus and these phenotype residuals can be evaluated by permutation (Abney 2015; Noble *et al.* 2017).

The composition of the strains phenotyped in a *C. elegans* GWA experiment can also impact the mapping power and QTL resolution. As explained above, *C. elegans* wild strains can be grouped into those strains that harbor one or multiple of the chromosome-scale selective sweeps on chromosomes I, IV, V, and X or those strains that do not have any sweeps and have higher levels of genetic diversity. These two groups of wild strains should be mapped separately because the effects on QTL power and resolution are different. GWA mapping of swept strains will have higher power to detect QTL because variants have higher allele frequencies than in divergent strains, but the detected QTL will have poorer resolution because LD is more extensive than in divergent strains. By contrast, GWA mapping of divergent strains will have lower power to detect QTL because most variants are rare, but the detected QTL will have higher resolution because long-range LD is lower than in swept strains. Despite these challenges, numerous GWA mapping have identified and validated QTL across the *C. elegans* species.

The two other selfing *Caenorhabditis* species will enable powerful GWA mapping but with different caveats. *C. briggsae* has greater population structure than *C. elegans*, so traits correlated with the tropical or temperate clades (Thomas *et al.* 2015) will require GWA mapping within each strain subset to avoid false positive QTL. *C. tropicalis* also has a significant population structure (Noble *et al.* 2021b), and it also has lower levels of genetic diversity than the other two selfing *Caenorhabditis* species. It will be interesting to see whether GWA mappings in these two species will work as well as they have for *C. elegans*.

Other *Caenorhabditis* species that do not self present a distinct set of issues for GWA. Most of these outcrossing species have been refractory to inbreeding, and so the usual methods of genotyping a strain once and then measuring many identical individuals are not applicable. Genotyping and phenotyping individual worms are possible but sacrifice many of the advantages of the *Caenorhabditis* model. Another issue is that the worms have such high levels of diversity that it will be difficult to generate reliable genotypes using reference-based short-read mapping. However, given the exceptionally low LD in highly diverse outcrossing *Caenorhabditis*, mapping resolution could be at the gene or variant level, as observed in large population samples in yeast (Bloom *et al.* 2013, 2015; Albert *et al.* 2014). As single-worm approaches increase in prevalence, GWA mappings in these species will become more commonplace.

GWA mapping has been performed for a variety of traits. In many cases, QTL have been narrowed to QTGs and quantitative trait variants (QTVs) using techniques discussed below (Evans *et al.* 2021). In a recent study, the fraction of dauers formed after exposure to ascaroside pheromones was mapped to multiple QTL, including a QTL on the X chromosome (Figure 5B) where variation in the gene *srg-37* was found to underlie differences across a population of 168 wild strains (Lee *et al.* 2019). The wild strain collection at the *C. elegans* Natural Diversity Resource (CeNDR) can be measured for a phenotype of interest and then mapped using tools available on the CeNDR website or the Andersen lab GitHub page (https://github.com/AndersenLab/NemaScan). CeNDR and NemaScan (Widmayer *et al.* 2021) make GWA mapping accessible to a wide variety of *C. elegans* research groups.

In some cases, multiple alleles of the same gene present on different haplotypes could underlie natural variation in a population. GWA mapping will not detect this locus because the individual haplotypes oftentimes do not reach high enough allele frequency for detection, even if they would when considered collectively. An alternative GWA mapping approach, called burden mapping (Price *et al.* 2010; Zhan *et al.* 2016; Hahnel *et al.* 2018), aggregates variation in genes to make a gene-by-gene association test across the genome. Allelic heterogeneity at the gene level is controlled because this aggregation makes a single test per gene. This technique was crucial for mapping a QTL for benzimidazole resistance in *C. elegans* to the beta-tubulin locus *ben-1* (Hahnel et al. 2018). In this case, many wild strains have independent variants in the *ben-1* gene so standard marker-based GWA mapping did not detect this locus even though it is known that the gene expresses the target of this drug class. This approach is powerful, but effects of population structure, demography, LD, and allele frequency need to be tested before it can be more widely adopted. Such studies are underway and new tools should be available on CeNDR soon.

### Multi-parent RILs—mix of linkage and association mapping

Linkage mapping in *C. elegans* uses controlled crosses to achieve balanced allele frequencies, but resolution is limited by the number of meioses and genetic diversity is limited to the two founding strains. Association mapping samples much broader diversity and uses historical recombination to generate high resolution, but rare alleles account for much of the variation, and population structure introduces substantial statistical confounding. Multiparental populations (MPP) provide a compromise between linkage and association, potentially offering the best of both worlds (Figure 1). A typical MPP is generated by systematically crossing more than two inbred lines, increasing the genetic diversity beyond that found in typical RILs or RIAILs while keeping relatively balanced allele frequencies and minimally structured populations to achieve better results than possible with conventional association mapping. MPPs have become important tools for model-system quantitative genetics, with powerful panels such as the Collaborative Cross and Diversity Outbred mice (Churchill *et al.* 2004, 2012), the *Drosophila* Synthetic Population Resource (King *et al.* 2012), the *Arabidopsis* Multiparent Advanced Generation Intercross (MAGIC) lines (Kover *et al.* 2009), and many more in crop species (Scott *et al.* 2020). The *C. elegans* community has generated two MPP panels, the four-parent mpRILs (Snoek *et al.* 2019) and the 16-parent *C. elegans* Multiparent Experimental Evolution (CeMEE) panel (Noble *et al.* 2017). Although MPPs are sometimes studied as genetically segregating populations, where each animal has a unique and partly heterozygous diploid genome, we typically let *C. elegans* populations go through a period of intercrossing and then derive inbred lines by selfing, as in the construction of RIAILs. The biology of *C. elegans* is such that heterogeneous populations are most conveniently studied by bulk-segregant analysis (BSA), as described in a later section, at least for questions about additive genetic effects. However, *C. elegans* biology is spectacularly suited to the construction of MPP-derived RIAILs; we can reduce LD to our hearts’ content by adding more generations of intercrossing, inbreed to homozygosity without major losses of fitness, cryopreserve the lines at minimal expense, and do it all in a matter of months rather than years.

MPPs offer new routes to the discovery of functionally important variation, and they raise new statistical challenges. To facilitate progress in MPP analysis, the Genetics Society of America created a portal to collect papers on these themes (https://www.genetics.org/content/multiparental_populations) (de Koning and McIntyre 2017). As always, experimental designs for MPP panels face tradeoffs. More founders means a broader sampling of diversity but at the cost of QTL detection power, as each founder haplotype becomes rarer as founder number is increased. In addition, more founders increase the likelihood that some strains will have genetic incompatibilities that can skew allele frequencies and decrease power. MPP panels offer improvements in mapping resolution if the founding lines share alleles but have deep histories of recombination that have rendered them independent; that is, the MPP can jointly leverage recombination events during strain construction and ancestral recombination events that occurred in the wild ancestry of the founder strains. Overall, more founders means better resolution at common alleles and lower power at uncommon ones.

Broadly speaking, there are two main modes of analysis for panels of MPP inbred lines: one can test for associations variant by variant, in the manner of an association analysis, or one can test for association with genomic segments according to which founding strain they derive from, treating the panel strains as mosaics of the founding inbred lines’ genomes (*e.g*., Broman *et al.* 2019). If, for example, gene-sized chunks are not recombined, then the gene-sized haplotype of each founder can be considered a unique allele, and the population thought of as potentially carrying an allelic series at each gene (Crouse *et al.* 2020). This approach allows for the variants within a haplotype to interact epistatically, conferring a unique phenotypic effect, even though the individual variants might also occur in different combinations in other founder haplotypes. At the same time, approaches that treat each founding strain’s haplotype as a unique allele may sacrifice statistical power, as the allele frequencies of each founder haplotype are lower than the allele frequencies of the minor allele at a single variant site that is shared among multiple founders. In other words, single-variant association is better for common variants and additive effects, and haplotypic approaches have advantages when intralocus epistasis is common.

The *C. elegans* mpRIL panel includes 200 inbred lines derived from four wild-type isolates from Orsay and Santeuil, France (JU1511, JU1941, JU1926, and JU1931). Jan Kammenga and colleagues systematically intercrossed the founders and their progeny for three generations and then inbred by six generations of selfing. A particular advantage of this panel is that it includes only natural genetic variation, uncontaminated by N2, offset somewhat by the fact that the founders are genetically similar to one another, and so some regions of the genome, such as the left side of chromosome II, are nearly invariant in the panel. Among the inbred lines, the frequencies of the four founder haplotypes are reasonably uniform across the genome, with a few exceptions, so that the panel retains substantial statistical power to detect allelic effects that derive from only a single founder. At the same time, the panel’s design offered little opportunity for recombination, and so the haplotype segments are long, similar to conventional RILs. Overall, the panel offers similar detection power to a RIL panel but with greater haplotypic diversity. Snoek and colleagues successfully mapped multiple QTL for lifespan and stress response traits, with an average QTL interval of 1.2 Mb (Snoek *et al.* 2019).

The CeMEE panel is the product of an unprecedented laboratory evolution experiment (Noble *et al.* 2017). Briefly, Henrique Teotonio and colleagues systematically intercrossed sixteen founding strains, including N2 and CB4856, over many generations to produce a highly outbred heterogeneous population. (Two pairs of founding strains are nearly identical, and so the population effectively descends from 14 separate founders.) They then subjected this population to 140 generations of experimental evolution under ordinary laboratory conditions. The populations were maintained on multiple large plates, keeping census size in the range of 10,000 animals. Each generation ended with a bleach treatment, enforcing four-day nonoverlapping generations. The researchers then derived hundreds of inbred lines from the evolved population, which is called the A6140 population. They generated still more inbred lines after another 50 generations of experimental evolution and then again after 50 more generations. These efforts produced more than 700 inbred lines, described as A6140 RILs, CA50 RILs, and CA100 RILs. The C and A in these appellations stand for “Control” and “Androdioecious,” to distinguish them from the GA50, GT50, and GM50 populations and RILs. Prior to inbreeding, these panels went through 50 generations of experimental evolution under gradually (“G”) increasing salt content in the plate media, after having had their mating systems altered to Trioecy (“T”) or Monoecy (“M”) via bulk introgression of *fog-2* or *xol-1* mutations. The *fog-2* allele converts hermaphrodites to females, and incomplete introgression resulted in a population with three sexes, males, females, and hermaphrodites. After the experimental evolution, the GT RILs were derived by inbreeding hermaphrodites. The GM population, incapable of outcrossing because *xol-1* males are inviable, experienced a rapid loss of diversity and the resulting inbred lines are of little value for mapping. The remaining collection of more than 1000 RILs derived from A6140, CA50, CA100, GA50, and GT50 makes CeMEE an unparalleled resource for genetic mapping. Noble and colleagues have genotyped the lines by sequencing and imputation and have carefully studied its mapping potential by simulation (Noble *et al.* 2017; 2021a).

The key feature of CeMEE is its low LD: the combination of ancestral and laboratory-generated recombination has shuffled the alleles, leaving variants largely uncorrelated with one another. The result is excellent mapping resolution. In a simulation study considering the 763 strains in CeMEE v2, QTL were found that explain as little as 3% of phenotypic variance have expected association confidence intervals on the order of 10–20 kb on chromosome arms and 50–100 kb on chromosome centers, with the association peak falling within 2.5 kb of the true causal variant across this whole set of parameters (Noble *et al.* 2021a).

The design of CeMEE also engenders some costs for mapping. Higher resolution trades off with detection power, so a larger number of lines is required to achieve the same ability to discover a QTL at a given genome-wide significance level. The long phase of experimental evolution also resulted in a spread of allele and haplotype frequencies, due to both drift and selection. Thus, many of nearly 400,000 variants segregating in CeMEE are at low frequency, and so can only be detected as QTL if their effect sizes are larger than would be necessary in a biparental RIL panel. The laboratory haplotype of *npr-1*, on the left side of the X chromosome, swept to fixation early in the history of CeMEE, eliminating all variation and mapping ability across more than a megabase. In addition, new mutations that arose during experimental evolution may contribute to phenotypic variation among the lines; more than 10,000 such mutations are present in at least three lines, and those at slightly higher frequency can be mapped as causal variants. Finally, the panel retains some population structure, so computationally expensive mixed-model approaches, similar to those used for association mapping in CeNDR, are required (Noble *et al.* 2021a). Tools for analysis of CeMEE data are available at https://lukemn.github.io/cemee/.

MPP panels are well suited to *Caenorhabditis* and we expect that they will play a sizable role in QTL mapping in the future. Some new multiparent populations are already in the works. To study outcrossing species, the Rockman lab has constructed an MPP-RIAIL panel in the obligate outcrosser *C. becei*, chosen because it tolerates inbreeding better than most such species. The panel derives from one male and two female wild founders, not inbred lines, and so the genetic diversity is derived from six founding genomes. Outcrossers are expected to harbor much more recessive deleterious variation than selfers. Conventional analysis of inbred lines is unable to distinguish recessive from additive effects, as only homozygotes are studied, but crosses between characterized inbred lines generate reproducible heterozygous genotypes and provide access to recessive variation. The experimental design that evaluates the F_1_ progeny of RILs or RIAILs is known as a recombinant inbred intercross (RIX) (Hua *et al.* 2003; Zou *et al.* 2005; Yuan *et al.* 2011). By crossing pairs of inbred lines reciprocally, researchers can use RIX designs to map maternal- and paternal-effect QTL separately from QTL that act zygotically.

Finally, we note that additional MPP designs have been successfully applied to quantitative genetics questions in other species but have not yet been applied to *Caenorhabditis*. One of the most ambitious and informative quantitative genetic programs is the nested association mapping (NAM) population in maize (Buckler *et al.* 2009; McMullen *et al.* 2009; Gage *et al.* 2020). The NAM design involves a collection of multiple RIL panels, each sharing one founder in common. QTL mapping within each RIL panel offers all the detection-power advantages we associate with RILs, but comparisons across the RIL panels provide the benefits of greater diversity and historical recombination that we associate with association mapping. A drawback is the exceptional size of a NAM population—the maize panel includes 5000 RILs. A similar MPP experimental design implemented in yeast, with each of 16 strains crossed to each of two others, includes 13,950 strains (Bloom *et al.* 2019). Phenotyping on massive industrial scales is required to gain the full benefits of these designs.

### Near-isogenic lines—a Mendelian approach to QTL mapping

Linkage and association mapping test for an effect of a variant by randomizing the rest of the genome. When an effect is detected, it reflects the average effect of the variant across the collection of randomized genetic backgrounds. An alternative approach to mapping is to simply eliminate all background variation instead of randomizing it. The key tool in this approach is the NIL (Eshed and Zamir 1995), a strain that carries a small region from one strain (the donor) introgressed into the genome of another strain (the recurrent parent) (Figure 1). Any phenotypic difference between the NIL and the recurrent parent is caused by an allelic difference within the introgressed region. NILs are sometimes called congenic strains, particularly in the rodent literature, but the *C. elegans* community has adopted the NIL vocabulary from plant genetics, where NILs have long been a key tool for genetic analysis. An introgressed region can be studied just as a classical allele would be, and indeed standard worm nomenclature guidelines provide an allele-naming convention—allele numbers are prefixed with IR (introgressed region), and donor and host are described with an arrow. For example, a strain that carries the CB4856 *npr-1* locus (and surrounding region) on the X chromosome introgressed into N2 is described as carrying *qgIR1(CB4856 > N2 X)*.

NILs are widely and effectively used to validate the locations of variants mapped by linkage or association, as we describe below in the “validation” section. Here, we describe the utility of NILs for genetic mapping. Given wild isolates that differ with respect to a trait of interest, QTL can be localized by surveying a panel of NILs that carry segments of one strain in the other strain’s background. Panels of NILs come in many flavors. The simplest is a panel of chromosome substitution strains (CSSs), in which each strain is homozygous for the recurrent parent genome except for a single chromosome, which comes entirely from the donor strain. A widely used *C. elegans* panel carries CB4856 chromosomes in the N2 background (Glauser *et al.* 2011; Evans *et al.* 2020). By surveying seven strains (N2 plus six CSSs), chromosomes that carry QTL can be identified (*e.g*., Glater *et al.* 2014). CSSs are manufactured by repeated backcrossing into the recurrent parent background, with genotyping at each generation to preserve donor DNA only on the chromosome of interest. Other mapping panels have been generated by recurrent backcrossing without genotyping, an approach that yields random segments of the introgressed genome, notwithstanding the effects of recombination and selection. The blind introgression approach can be tailored to target particular parts of the genome by using an existing NIL (or RIL or RIAIL) as the donor strain. The most widely used NIL panel for *C. elegans* was generated by recurrent backcrossing of several N2/CB4856 RILs into the N2 strain, yielding a collection of 90 strains, each with a single segment of a few megabases of CB4856 genome in the N2 background (Doroszuk *et al.* 2009). The strains collectively tile across 96% of the genome, and NIL collections of this type are called tiled NILs (Figure 1). The Kammenga lab has also constructed a reciprocal panel, with N2 segments introgressed into the CB4856 strain (Sterken and Kammenga, personal communication). A third design, after CSSs and tiled NILs, is stepped NILs, a panel of NILs that all share one introgression boundary and vary only at the second boundary (Koumproglou *et al.* 2002). In other words, the introgressions are nested, each containing slightly less donor material than the last. These strains can be generated by starting with a single NIL and selecting recombinants (with or without the help of visible markers) that break the introgressed region into smaller and smaller chunks. Bernstein and Rockman generated and genotyped 870 stepped NILs that step through a 1.4 Mb region of the X chromosome (Bernstein and Rockman 2016).

Introgression-based mapping is an important tool in studies of reproductive incompatibility. Donor regions that cannot be introgressed into a recurrent parent are incompatible with that strain’s genetic background, and so missing tiles in a NIL library might reveal incompatibilities. Bi *et al.* used marker-facilitated introgression to identify regions of the *C. briggsae* genome that were incompatible with the genome of its sister species, *C. nigoni* (Bi *et al.* 2015). Even when NILs are viable, they may show directional declines in fitness consistent with incompatibilities (Snoek *et al.* 2014), and crosses among them may reveal higher-order interactions that contribute to speciation (Bi *et al.* 2019). This growing line of research in *Caenorhabditis* parallels similar applications in *Drosophila* and tomato speciation genetics (Masly and Presgraves 2007; Moyle and Nakazato 2009).

NIL-based QTL mapping has some important caveats. First, NIL mapping abandons the statistical efficiency of linkage and association mapping, retreating instead into the rigorous logic of Mendelian genetics. The result is that sample size requirements are quite different (Keurentjes *et al.* 2007). To detect an effect of a given size, linkage and association can pool data from many genetically different strains. With NILs, analysis typically involves pairwise comparisons of strains, and consequently, detection power scales with the number of replicates per strain and not with the number of strains. A corollary of pairwise comparisons is that background mutations (that is, mutations that arise during strain construction, as well as small, unobserved introgressed segments) are completely confounded with the introgressed interval, exactly as they are with alleles derived from mutagenesis. Background mutations are less of a concern with linkage mapping and association, where such mutations are unique to individual strains and contribute statistical noise but not confounding. Second, effects of donor segments are detected in a single recurrent parent genetic background, eliminating background genetic variation. The single background makes small-effect variants easier to discover (Eshed and Zamir 1995; Shao *et al.* 2008), but it also means that the additive effect of the donor segment is completely confounded with epistasis between recurrent parent and donor alleles. In some cases, this is a desirable outcome, converting hard-to-detect epistatic variance into easy-to-detect additive variance, but in other cases it will cause the concealment of variants whose effects are masked by the recurrent parent background. NIL QTL effects will therefore not always closely match the additive effects of alleles averaged over backgrounds, and they could reflect idiosyncratic or even pathological epistatic interactions. For example, for the interaction shown in Figure 2, each locus would show a large effect in the AB1 recurrent-parent background but none at all in the N2 recurrent-parent background. Epistatic effects are likely to be quite common in selfing species like *C. elegans* (Dolgin *et al.* 2007; Snoek *et al.* 2014), and when using NILs for validation, researchers typically account for such effects by assaying reciprocal NILs, with the focal region from each strain introgressed into the other strain.

The analysis and interpretation of NIL data differs in important ways from that of linkage or association data. Despite its statistical inefficiency, pairwise comparison between strains is the safest method of analysis. Methods that test marker effects averaged over strains, as in linkage or association mapping, are likely to work well for traits affected by a small number of large-effect loci, such that NILs can be divided into a few phenotypic classes; approaches such as “bin mapping” (Doroszuk *et al.* 2009) work in this case. For traits with more complex genetic architectures, particularly polygenic or epistatic architectures, marker (or segment) regression approaches are prone to a variety of artifacts, caused by the idiosyncratic LD structure of NIL panels. This problem is particularly acute in stepped NILs, where alleles at opposite ends of the maximal introgression almost always have opposite strain origins, generating strong LD.

Given that pairwise contrasts are the safest analysis method, the question becomes, which strains should we contrast? Statistically testing all pairs of strains imposes a large multiple-testing burden, and the results are hard to interpret. One classical approach is called the common segment method (Snell and Bunker 1965; Shao *et al.* 2008). Each NIL is tested for a difference with the recurrent parent, which carries no introgression. If strains that differ from the recurrent parent have overlapping introgressions, the smallest overlap is inferred to carry a QTL. Strains that carry the QTL but do not differ from the recurrent are inferred to also carry suppressors. This approach is fairly *ad hoc*, and it infers some QTL (suppressors) from the presence of nonsignificant tests, something that makes statisticians wince. An alternative approach involves sequentially testing each pair of most-similar strains, testing the minimum number of strain pairs to achieve the greatest possible mapping resolution. Shao *et al.* (2010) developed an algorithm to find the best set of contrasts, constructing a minimum spanning tree of NIL genotypes and then testing adjacent pairs along the branches of the tree. In several studies of *C. elegans* traits, this sequential mapping technique has identified suites of QTL (Green *et al.* 2013; Glater *et al.* 2014).

A common outcome of NIL analysis, in *C. elegans* as elsewhere, is the discovery of a large number of QTL, many more than are detected in analyses of RILs from the same founders and often with effect sizes whose sums greatly exceed the phenotypic difference between the founding strains (Shao *et al.* 2008). For example, a series of studies of dauer formation (Green *et al.* 2013, 2014) identified more than 30 QTL in NILs carrying CB4856 donor segments in the N2 background (Doroszuk *et al.* 2009). These findings reinforce the polygenic architecture of complex traits in *C. elegans* and the improvement in detection power afforded by NIL mapping. At the same time, the size of the effects in NILs and their invisibility in RILs suggests that many NIL-detected QTL are amplified by interactions with the fixed genetic background (Shao *et al.* 2008). Bernstein and colleagues (Bernstein *et al.* 2019) pushed this question to its limits by studying population growth-rate phenotypes in stepped NILs that subdivide a single 1.4 Mb region of the X chromosome into 15 small intervals, each encompassing about 0.1% of the genome. They detected QTL in nine of the 15 intervals. The effects of adjacent QTL were in opposite directions, so that tight linkage between them masked their effects in their native contexts. This pattern of linkage masking (Brown *et al.* 2016) is emerging as a potentially important mechanism by which segregating variation is masked in populations (Schoech *et al.* 2020).

### Bulk-segregant analysis enables high-resolution QTL detection

All of the quantitative genetic mapping procedures described so far use strain resources that can be shared among labs, including RILs, wild isolates, or NILs. The creation of these resources requires investments of time and money to generate the strains and to genotype them. In addition, the strain sets and mapping approaches we have presented so far do not use selection to identify or enrich for phenotypically different individuals. In this section, we will present BSA, which does not require the generation of strains. In this mapping procedure, a researcher experimentally generates a population of genetically heterogeneous individuals. One then compares allele frequencies among individuals with the phenotype of interest to allele frequencies in an unselected pool or a pool of individuals with the opposite phenotype. Isolation of the desired phenotypic class can involve worm-by-worm measurements by the researcher or mass selection imposed by relevant environmental perturbations. Unlike the other mapping methods, phenotype distributions draw from a genetically heterogeneous pool of individuals, including heterozygotes, and consequently dominance can influence the detection of QTL.

For more than 20 years, BSA mapping has facilitated the identification of many mutant genes in the laboratory-adapted strain N2. These bulk-segregant analyses take an N2 mutant of interest and cross it to a genetically divergent strain to generate a heterozygote. After allowing the heterozygote to self, the experimenter selects for the mutant phenotype of interest in the next generation. The individuals with the mutant phenotype will carry N2 regions of the genome linked with the mutation of interest but unlinked genomic regions will be from either N2 or the genetically divergent strain in Mendelian proportions. Regions of the genome linked but distant from the mutation will recombine with the genetically divergent strain. Individual recombinants will have unique combinations of the genetically divergent genomic regions except where the mutation is located, which will have N2 markers. The more recombinants that are scored the more precisely localized the mutation of interest will be. These regions are identified by genotyping either restriction fragment polymorphisms (Davis *et al.* 2005), SNVs from reduced representation sequencing (O’Rourke *et al.* 2011), molecular inversion probes (Mok *et al.* 2017), or whole-genome sequencing (Doitsidou *et al.* 2010). Allele frequency skews in the N2 direction suggest linkage with the mutation of interest. In practice, observed allele frequency skews could also be caused by experimental bottlenecks that lead to fewer recombinants generated in some intervals. In addition, genetic incompatibilities between the two strains can cause allele frequency skews in this cross design, as has been observed previously (Ben-David *et al.* 2017). Epistatic interactions between the focal mutation and the genetically divergent strain can also cause skews toward N2 at sites not linked to the mutation, as only strains that carry both the mutation and the potentiating background will be selected. In some cases, these accidental skews have been used to map the interactors (Noble *et al.* 2020).

In the context of natural variation, phenotypically divergent strains should be identified and then crossed in the same way that N2 and genetically divergent strains have been in the past. If the two wild strains have few genetic differences, then it might be easier to identify the specific variant(s) underlying the phenotypic difference, as has been done with laboratory strains N2 and LSJ2 (McGrath *et al.* 2011; Large *et al.* 2016; Zhao *et al.* 2020). Oftentimes, phenotypically divergent strains are also genotypically divergent, so many variants must be evaluated for linkage with the trait variation. In *C. elegans*, nearly all extant wild isolates have genome sequence available through the *C. elegans* Natural Diversity Resource (Cook *et al.* 2016b), so design of genotyping strategies is straightforward. From simulations and empirical work, it has been shown that QTL can be identified by BSA using different strains and that QTL confidence intervals can be smaller than what are obtained using linkage mapping or NIL mapping approaches, especially after many generations of crosses to reduce LD (Burga *et al.* 2019). QTL detection power and mapping resolution are dependent on the intensity of selection (higher is better), number of recombinants generated (the more the better), the effect size of the QTL, and the genetic complexity of the trait. If these procedures can be scaled to map variation between many different pairs of genetically divergent strains, then BSA has the promise to identify QTL and causal genes that underlie variation for many quantitative traits. However, BSA is less efficient in several situations. First, genomic regions where recombination in limited (chromosome centers) lower the mapping resolution. Second, BSA depends on the strength of selection for the particular trait difference. It is not easy to select for the phenotypically extreme individuals from both sides of a phenotypic distribution in all traits. For example, it is difficult to select for sensitive individuals in drug responses because these individuals are dead or do not grow and can not be genotyped. BSA approaches for drug responses depend on selection for drug-resistant individuals. Third, genetic incompatibilities could make some strains unlikely or impossible to generate recombinant progeny (Seidel *et al.* 2008; Ben-David *et al.* 2017; Ben-David *et al*. 2021; Noble *et al.* 2021b). For example, BSA will have allele frequency skews in genomic regions where the two genotypes do not generate progeny in equal proportions because of effects on growth or reproduction, as has been observed in *C. elegans* and *C. tropicalis*. Fourth, the statistical power and resolution of QTL identified by BSA depend on the number of recombinants generated. With selfing species like *C. elegans*, self-progeny must be prevented to ensure that all the individuals in the next generation were generated from a cross with the genetically divergent strain, using a mutation in the gene *fog-*2 (Schedl and Kimble 1988) that feminizes hermaphrodites as done previously (P.T. McGrath, personal communication). Genome-editing approaches that delete the *fog-2* locus make selfing strains into obligately outcrossing strains offering an effective method to mitigate this issue. One can also use small pools of RILs in an assay similar to BSA to identify genomic intervals enriched in one phenotypic class (Frézal *et al.* 2018; Koneru *et al.* 2021). Overall, BSA offers a powerful method to connect phenotypic differences to specific genes using *C. elegans* and other selfing *Caenorhabditis* species. An R package was created to help with *C. elegans* BSA experiments (https://github.com/eyalbenda/xQTLstats).

BSA approaches can also make use of population-wide diversity by selecting bulk populations from the tails of a pool of wild isolates, an analog of GWA that allows ancient recombination to shuffle alleles, albeit imperfectly. In one study, starvation survival was selected in a pool of animals representing 96 wild strains (Webster *et al.* 2019). They used the allele frequency changes during the experiment to infer the relative survival rates for each of the 96 strains, and they then performed GWA on these inferred probabilities. The approach, which led to discovery of a starvation-resistance QTL on chromosome III, is equivalent to BSA analysis of the allele frequencies among the survivors, but by first inferring strain identities and then imputing genotypes genome-wide, it improves on the raw allele frequency estimates.

In outcrossing *Caenorhabditis* species, BSA offers one of the few methods to connect phenotypic variation to genotypic variation because levels of genotypic variation are so high. In *Caenorhabditis*, linkage, GWA, and NIL-based mapping all depend on genotypically defined strains, but most outcrossing *Caenorhabditis* species do not have defined strains because inbreeding depression limits the ability to make strains homozygous at all genomic loci. Therefore, isolates do not breed true from generation to generation. BSA mapping in these species tracks allele-frequency shifts after selection. With higher levels of variation and recombination, QTL detection will be more difficult because markers must be tightly linked to the causal locus, but once detected QTL resolution should be higher.

It is also possible to identify QTL using an evolve-and-resequence approach. In this approach, a genetically heterogeneous population is exposed to selection. This approach can use either directional selection, where only individuals with particular phenotypes are chosen to breed, or experimental evolution, in which the experimental environment imposes selection but the exact phenotypes are unknown. After some generations of evolution, allele frequencies in the evolved population are compared to those in their ancestors or in control populations subjected to similar numbers of generations without the selective conditions. This approach can identify QTL that vary within the founding population, as has been performed for stress responses using *C. remanei* (Sikkink *et al.* 2019). Experimental evolution is described at length in a previous WormBook chapter (Teotónio *et al.* 2017). Overall, BSA and its kin offer powerful approaches to identify the genetic variants that underlie phenotypic differences in selfing and outcrossing *Caenorhabditis* species.

### *Caenorhabditis* quantitative genetics from QTL to gene and variant

Quantitative genetics can teach us about the causes of evolutionary change and about the molecular mechanisms underlying important traits. A critical step is to connect a QTL to a specific gene and ultimately to the variant that causes the phenotypic difference. *C. elegans* has made this connection many times over the past 20 years because of distinct advantages in generation time, genome-editing tools, and inbred strains (Evans *et al.* 2021). These results have impacted our understanding of mating system evolution (Palopoli *et al.* 2008), genetic incompatibilities (Seidel *et al.* 2008, 2011; Ben-David *et al.* 2017), toxin responses (Zdraljevic *et al.* 2017, 2019; Brady *et al.* 2019; Evans and Andersen 2020; Na *et al.* 2020), and behavioral variation (de Bono and Bargmann 1998; McGrath *et al.* 2009; Reddy *et al.* 2009; Bendesky *et al.* 2011, 2012; Greene *et al.* 2016a, 2016b). In this section, we will describe the process to go from QTL to QTG and to QTV. Notably, all genetic mapping approaches, including the quantitative genetic mapping approaches described here, involve a trade-off between continuing to map to finer resolution *vs* moving to candidate gene testing. As we describe in the next section, any mapped interval should be confirmed using crosses before moving to candidate genes. Mapping provides positional candidate genes, and the transition from positional candidates to candidates with plausible or suggestive biological annotations is fraught with danger. The shortcomings of candidate-gene storytelling are well documented (Pavlidis *et al.* 2012), and mistaken assumptions about causal genes have sometimes caused whole fields to move in the wrong direction (Smemo *et al.* 2014; Claussnitzer *et al.* 2015). It takes genetic experiments to determine causal links between genetic variants, biological mechanisms, and phenotypes.

Additional experiments and mapping approaches can help narrow QTL to smaller genomic intervals and give higher confidence in the detection of QTL. For linkage, NIL, and BSA mapping approaches, additional lines or recombinants can increase confidence in correlated genotypic markers and add markers that are correlated with the trait difference. Once a QTL is detected, additional strains or recombinants with recombination breakpoints within the QTL interval (but not scored previously) can be used to narrow the QTL interval. This approach has been instrumental in narrowing broad QTL to small genomic intervals with fewer candidate genes (Zdraljevic *et al.* 2017, 2019; Evans *et al.* 2021). For GWA mapping, wild strains with novel haplotypes or haplotypes with breakpoints nearby the markers most highly correlated with the phenotypic difference can also better define the QTL. This process is limited by haplotype information and historical recombination across QTL identified by GWA. The combination of mapping results from different methods offers a powerful way to narrow QTL as well. Overlap between QTL identified using linkage and/or NIL and GWA analyses can be a powerful method to identify candidate genes. This technique works well when common variation is shared by one of the two founding strains used in the linkage, NIL, or BSA mapping. In some cases, single or a small number of candidate genes are identified. Once QTL are detected from initial mapping experiments, QTL can be narrowed by scoring additional strains and by comparing different quantitative genetic mapping methods. The ultimate goal is to identify one or a small number of candidate genes that can be tested for causal links between genotypic and phenotypic variation.

**Figure 6:**
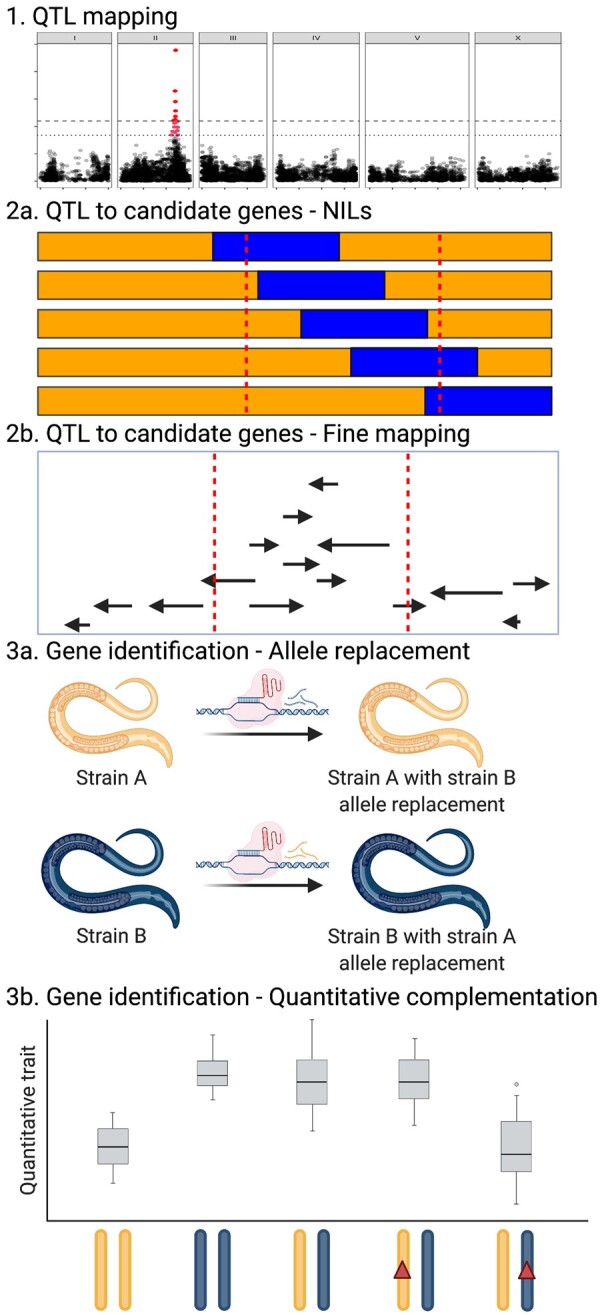
Genetic causality experiments in *C. elegans*. Step 1 is to identify QTL using statistical mapping approaches. A GWA mapping is shown here. Step 2a uses NILs to narrow the QTL confidence interval found in Step 1, shown as dotted red vertical lines. In parallel or iteratively, QTL fine-mapping approaches like Step 2b can also narrow a QTL to candidate genes. Once candidate genes are identified, genome-editing using CRISPR-Cas9, as shown in Step 3a, can replace the candidate gene allele from Strain A with the Strain B allele and vice versa. Sometimes candidate genes have many different variants or no single variant can be tested as in Step 3a. In these cases, a loss-of-function allele like a deletion shown in Step 3b can be created in both Strain A and Strain B genetic backgrounds using CRISPR-Cas9 genome editing. Then, these new deletion strains can be crossed to the reciprocal parent strains and compared to the parent strains and heterozygotes in a quantitative complementation experiment. In the example shown here, Strain A (orange) has a low trait value and Strain B (blue) has a high trait value. The Strain B allele confers a dominant phenotype as seen in the heterozygote. Deletion of the Strain A allele has no effect on the phenotype of the heterozygote but loss of the Strain B allele fails to complement the Strain A allele. These data indicate that the gene deleted is the same gene that confers a trait difference between these two strains.

All of the mapping approaches presented in this chapter are statistical measures of correlation between genotypic and phenotypic variation. Like any statistical method, mapping could identify false-positive QTL and miss true positive QTL. Experimental tests of specific genomic regions in isolation can mitigate many concerns about false-positive QTL. NILs offer a ready approach to both confirm that a QTL causes a phenotypic difference and to narrow QTL to candidate genes (Figure 6). One distinct advantage of *Caenorhabditis* species is that crosses are rapid and NILs can be easily generated between any two strains using insertion-deletion variants to genotype the interval and genetic background. CeNDR offers an indel finder and primer generator for any pairwise strain comparison to aid this process. The *C. elegans* N2xCB4856 RILs, RIAILs, and NILs have been used to identify numerous QTL that have been narrowed using existing or newly constructed NILs. Once a NIL recapitulates the observed phenotypic effect identified in the mapping experiment, this NIL can be repeatedly crossed to the recurrent genetic background to make increasingly smaller NIL intervals. This approach is limited by recombination distance and easy-to-genotype markers to detect recombination events within the interval. In mapping panels with more than two founders, including GWA and MPP, the choice of NIL parent strains is not obvious because numerous strains with different haplotypes in the QTL region harbor the reference or alternative genotype at the peak QTL marker. In addition, parent strains should be chosen with known incompatibilities on chromosomes I and III taken into consideration (Seidel *et al.* 2008; Ben-David *et al.* 2017). Genetically compatible strains will be easier to make NILs if they are reproducibly phenotypically divergent and have opposing genotypes at the peak marker in the QTL. Once NILs are identified or created, then the same quantitative assay that identified the QTL should be performed to confirm and narrow the QTL interval. The identification of a genotype-phenotype correlation, as with NIL mapping, can be repeated iteratively with ever-smaller NILs to narrow the interval to candidate genes. The efficiency of this process is a distinct advantage of quantitative genetic mapping in *C. elegans*. Once a QTL is detected, genetic crosses to make NILs are trivial and reduce the dependency on mapping alone to define candidate genes. However, QTL effects can fractionate to smaller effect QTL, as observed previously (Glater *et al.* 2014; Evans *et al.* 2018; Bernstein *et al.* 2019), so iterative cross approaches should be cognizant of this limitation.

NILs can narrow QTL intervals to lists of candidate genes that can be tested using quantitative phenotyping assays. Candidate genes within a QTL interval need to have variation to underlie the phenotypic differences observed in the mapping population or cross. For this reason, scans of variation in coding regions or upstream gene regulatory regions should be performed to make candidate gene lists (but keep in mind that regulatory variants can be distant from the gene they regulate, even in *C. elegans*, Bendesky *et al.* 2012). Coding variation that is predicted to affect function is an easy starting point, but gene expression variation can be equally impactful. In many cases, many noncoding variants surrounding candidate genes exist so connections to the gene of interest are not easy to make computationally. In the context of QTL between the N2 and CB4856 strains, researchers can take advantage of previous work mapping alleles that affect transcript abundance in RILs and RIAILs (Li *et al.* 2006; Rockman *et al.* 2010; Viñuela *et al.* 2010; Francesconi and Lehner 2014). Genes with large-effect local (likely *cis*-acting) regulatory variation are known and can be prioritized for follow-up. Importantly, CeNDR contains single-nucleotide variants (SNVs) and insertion-deletion (indel) variants less than 50 bp, but additional variation could impact gene function and is not curated across *C. elegans* strains yet. New data, algorithms, and simulations to validate variant calls are required to generate lists of these other variants, including short-tandem repeats, transposon insertions and excisions, large chromosomal aberrations (inversions, deletions, duplications, translocations), and copy-number variants. These types of variants can underlie differences in gene functions for quantitative traits as seen in *C. elegans* previously (Palopoli *et al.* 2008; Zhao *et al.* 2020), but each requires additional analyses.

Once candidate genes are identified, then these genes can be tested for a causal role in trait variation. Just like in molecular genetics experiments to establish genetic causality, complementation tests using existing mutant alleles can work but genetic background differences between the two strains in the complementation test can be problematic. In addition, one should not assume that the N2 allele of a gene is wild-type (*e.g.*, *npr-1*Sterken *et al.* 2015), so other methods might be preferred. Molecular geneticists might test “complementation” using transgenic overexpression of the N2 wild-type version of gene. This method might work for natural variants, but the caveats about genetic background, reference genome bias, and overexpression can be problematic. When coding variation caused by a single SNV or indel is implicated, then CRISPR-Cas9 genome editing can readily change each parent strain to contain the single variant site from the other parent strain. These reciprocal allele replacement strains when compared to the parent strains should alter phenotypes to match the genotype at the QTN. Given the ease of genome editing in *C. elegans*, this method is preferred to complementation-based assays. If the candidate gene has many variants, then a simple single allele-replacement experiment will not suffice. One strain might have lost the function of that gene compared to the strain that still maintains that gene function. CRISPR-Cas9-induced gene deletions are predicted to cause different effects in these two genetic backgrounds in this case. In the parent strain that has lost the function of the gene, a deletion of that same gene should not alter the phenotype. By contrast, a deletion of that gene in the strain that maintains the function of that gene should cause a phenotype similar or equivalent to the strain without the function of the gene. To ensure that alteration of the phenotype is caused by the specific gene deletion, reciprocal hemizygosity tests (Bendesky *et al.* 2012; Stern 2014; Turner 2014; Brady *et al.* 2019) can be performed if the quantitative phenotype is recessive. In this experiment, the deletion of the gene in one genetic background is crossed to both the same and the other genetic backgrounds. This procedure is repeated for deletion of the gene in the other genetic background. Two of these four strains are homozygous at all loci except for the candidate gene, and the other two of the four strains are heterozygous at all loci except for the candidate gene. When compared to the two homozygous parent genetic backgrounds, the effects of the candidate gene can be tested explicitly. This procedure has identified the gene underlying several QTL in *C. elegans* (Evans *et al.* 2018, 2020, 2021; Brady *et al.* 2019; Evans and Andersen 2020). The ease and speed of *C. elegans* genetics and genome editing make gene causality testing possible and a powerful approach to identify QTG and QTN underlying phenotypic variation in the species.

For most *C. elegans* geneticists, it is tempting to perform RNAi by feeding to test candidate genes in traits of interest. This approach requires caution for studies using wild strains because of natural variation in the RNAi response (Tijsterman *et al.* 2002; Pollard and Rockman 2013; Paaby *et al.* 2015), though with appropriate controls RNAi can still be informative (Noble *et al.* 2015; Paaby *et al.* 2015; Vu *et al.* 2015; Torres Cleuren *et al.* 2019).

## Evolutionary lessons

Quantitative genetic studies of *C. elegans* started in the 1990s, more than half a century after similar work began in *Drosophila* and mouse. Despite that late start, worm researchers have wrangled some fundamental lessons about the forces that shape heritable phenotypic variation.

The first discovery and an early lesson is that domestication imposes powerful and sometimes surprising patterns of selection. *C. elegans* domestication involved a single-worm bottleneck followed by hundreds of generations of evolution under novel conditions, and the result is that evolution was dependent on new mutations, often with very large effects (Sterken *et al.* 2015). Many of the nearly Mendelian variants discovered in *C. elegans* quantitative genetics have proven to be recent mutations that arose during the 20th century during adaptation to laboratory conditions.

A second discovery is that the genomic distribution of QTL is strongly shaped by selection on traits unrelated to those under study. In other words, the probability that a gene carries phenotypically relevant variants is strongly influenced by selection on the genes nearby. The linked selection can be positive, with advantageous mutations sweeping through the population (selective sweeps), or purifying, with deleterious mutations eliminated from the population (background selection). In either case, selection on a focal site eliminates genetic variation from a population over a large region of a chromosome, with the exact extent dependent on the details of the selective effects and the rate of recombination. In *C. elegans*, background selection gives all of the chromosomes a characteristic pattern of low genetic variation in the gene-dense chromosome centers (Rockman *et al.* 2010), and selective sweeps have stripped the bulk of genetic variation from huge sections of four different chromosomes (Andersen *et al.* 2012; Lee *et al.* 2021). These patterns are visible in the distribution of DNA sequence variation, but they also manifest at the level of quantitative genetic variation, as for example in the distribution of genes whose transcript abundances are affected by nearby variants (Rockman *et al.* 2010). The effects of selection at linked sites depend on the population-effective recombination rate, which involves both the per-meiosis crossover frequency (one per chromosome in *C. elegans*) and the frequency with which individuals are heterozygous at multiple sites. The extremely high rate of selfing in *C. elegans* amplifies the effects of selection at linked sites by decreasing the effective recombination rate.

A third lesson, illustrated in part by the effects of linked selection, is that the mating system has profound effects on nearly every aspect of quantitative variation. The partial-selfing mating system of *C. elegans*, *C. briggsae*, and *C. tropicalis* is starkly different from the random mating of idealized Wright-Fisher population genetics. Although worm geneticists often think of *Drosophila* geneticists as our closest kin, for quantitative genetics our touchstones should be in the plant genetics world, particularly in species with mixed-mating systems, such as *Arabidopsis lyrata*, *Capsella rubella*, and *Mimulus* species. The transition to selfing in *Caenorhabditis* lineages is associated with a dramatic increase in homozygosity, presumably associated with a purging of large-effect recessive deleterious variants and possibly the fixation of weak-effect variants. A side effect of low rates of outcrossing and recombination is that whole genomes evolve as units, and alleles are rarely tested in new combinations. An expected effect is outbreeding depression, where matings produce F_2_ individuals that carry shuffled genomes that suffer from mismatched alleles. Evidence for outbreeding depression is extensive in all three selfing species (Dolgin *et al.* 2007; Gimond *et al.* 2013; Cutter *et al.* 2019). Low rates of recombination allow for coadaptation among loci, increasing the likelihood that epistatic interactions contribute to trait variation (Gaertner *et al.* 2012). For traits under stabilizing selection, each inbred isolate may have ended up with a unique genetic solution, and crosses therefore reveal abundant transgressive segregation. This kind of population history also facilitates the accumulation of cryptic genetic variation, alleles that are invisible in their usual setting but available to fuel adaptation to novel conditions or for discovery in QTL-mapping studies.

One surprising finding from quantitative genetic studies in *C. elegans, C. briggsae*, and *C. tropicalis* is that each species carries a distinctive and unusual type of post-zygotic gene drive element. These haplotypes express an offspring-killing toxin in the gametes (usually the oocyte but in one case the sperm) and an antidote that is expressed in the offspring. In an animal heterozygous for such a haplotype, self-fertilization results in delivery of the toxin to 100% of the offspring but the antidote only to 75%. This class of gene drive element, known broadly as a Medea element, is very rare outside of these three species. Because the offspring-killing only happens in heterozygotes, and these species mostly self, the evolutionary causes and dynamics of Medea elements in *Caenorhabditis* are quite mysterious. An important effect is increased outbreeding depression, though here the cause is interaction between parental and offspring genotypes at a single locus, and not the expected interaction between loci newly shuffled by recombination.

Finally, *C. elegans* quantitative genetics carries lessons about the genetic architectures of complex traits. Mendelian effects appear largely limited to recent lab adaptation and to male-related traits. Most QTL mapping has focused on narrowly defined phenotypes, often with unclear relationship to fitness in the wild, and these have been the most productive for the method. Often a handful of relatively large effect loci are discovered, and experimental fine-mapping and validation has led to new findings about the biology of a range of topics. Other traits have proven to be extremely polygenic, such that no individual locus emerges as significant in linkage or association mapping, though methods tailored to polygenic effects can show that they exist (Noble *et al.* 2017). The range of genetic complexities in *C. elegans* mirrors that found in other experimental systems, including humans. After all, man is but a worm.
